# Entropy Drives the Predictive Discovery of an Optimal Cleavable Comonomer for ROMP

**DOI:** 10.1021/acscentsci.5c00521

**Published:** 2025-07-23

**Authors:** Kwangwook Ko, Piper L. MacNicol, Mingming Zhu, Lei Zhang, Saifudin M. Abubakar, Jeremiah A. Johnson

**Affiliations:** † Department of Chemistry, 2167 Massachusetts Institute of Technology, 77 Massachusetts Avenue, Cambridge, Massachusetts 02139, United States; ‡ ExxonMobil Asia Pacific Research and Development Co., Ltd., Shanghai 200241, China; § ExxonMobil Asia Pacific Pte. Ltd., 1 HarbourFront Place, Singapore 098633, Singapore

## Abstract

Ring-opening metathesis polymerization (ROMP) of norbornene derivatives enables access to polymeric materials for applications ranging from targeted drug delivery to high-performance thermosets; however, the carbon–carbon backbones of ROMP-derived poly­(norbornenes) resist deconstruction under mild, selective conditions. Cleavable comonomers (CCs) have been introduced to address this limitation, yet their implementation has been hindered by prohibitive costs and/or suboptimal reactivity. Moreover, the discovery of existing CCs has been largely empirical, lacking clear design principles. Here, we identify the entropy of ring-opening as one of the key determinants of ROMP copolymerization behavior of the best-performing CCs reported to date. Guided by this insight, we establish predictive design criteria and introduce **Me**_**4**_ **Si**_**2**_ **O9**, a CC that exhibits near-ideal room temperature copolymerization with a broad range of norbornene-based (macro)­monomers. **Me**_**4**_ **Si**_**2**_ **O9** is significantly less expensive than leading silyl ether-based CCs and enables uniform incorporation of cleavable linkages into polymer backbones at low loadings. Beyond delivering a cost-effective and high-performance CC, this work provides fundamental insights into ROMP copolymerization that will enable predictive CC development and expand the functional scope of deconstructable polymeric materials.

## Graphical abstract

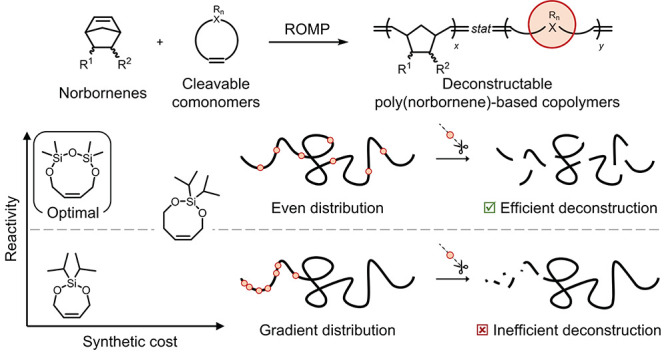

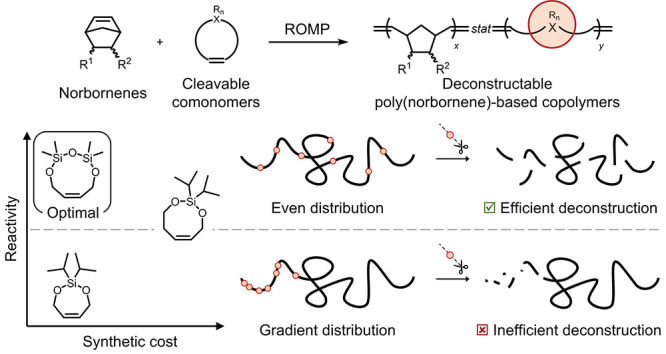

## Introduction

The development of deconstructable polymers, i.e., polymers that can undergo selective backbone cleavage reactions to form products with defined chemical functionality, has garnered considerable attention, motivated by applications ranging from sustainable materials to drug delivery.^1–10^ An effective strategy for introducing chemical deconstructability to polymers involves integrating cleavable comonomers (CCs) into the polymer backbone via copolymerization, thereby embedding functionalities that can be selectively cleaved by external stimuli.^11–22^

Imparting deconstructability can often be achieved without compromising the specific thermal, mechanical, or chemical properties that polymers are designed to possess, which is crucial for maintaining the function of polymers in their intended applications. In the context of CCs, a common strategy to achieve this objective is to limit the CC loadings to low levels,^11–15,17^ thereby minimizing disruptions to the polymer’s intrinsic characteristics while also helping to control overall costs. Under such constrained loading conditions, the copolymerization reactivity of the CC becomes particularly critical, as the CC must uniformly integrate into the polymer chains to enable efficient deconstructability. Yet, developing CCs with optimal reactivity remains highly demanding, with only a few successful examples reported to date.^14–16^ Moreover, even at minimal loadings, the CC must remain cost-effective; a prohibitively expensive CC negates the economic advantages of low loading and limits practical applicability. Despite notable advancements, most—if not all—current CCs fail to meet these stringent requirements of both optimal reactivity and affordability.

Ring-opening metathesis polymerization (ROMP) is a versatile technique widely employed for the synthesis of polymers with diverse properties and architectures.^23–28^ The ROMP of strained norbornene derivatives (NBEs) is particularly effective for producing materials with broad applicability, ranging from drug delivery systems^29–31^ to engineering plastics;^32–35^ however, the exclusively carbon-based backbones of these polymers presents intrinsic limitations to deconstructability, underscoring the potential for suitable CCs to address this limitation. Cyclic olefin monomers with varied designs have been developed for the synthesis of deconstructable ROMP-derived polymers,^11–13,21,36–52^ yet only a few studies have focused on low loading systems (i.e., at loadings of 10 mol % or lower),^11–13^ where the properties of the original material are mostly retained. Even in those instances, practical application is hindered by challenges such as prohibitive synthesis costs and limited deconstructability (Figures 1A and 1B). For instance, although the silyl ether-based CC **iPr**_**2**_ **Si8** and acetal-based CC **iPrAc8** exhibit good reactivity with NBEs, their synthesis relies on relatively expensive starting materials. By contrast, their 7-membered ring analogs, **iPr**_**2**_ **Si7** and **iPrAc7**, are cheaper but suffer from relatively poor reactivity.^13,21^ Dihydrofuran (DHF) presents another economical alternative;^12,39^ however, it significantly retards ROMP by forming stable Fischer carbene species, thereby limiting monomer conversion in polymerizations targeting moderate to high degrees of polymerization (DP). Moreover, the tendency of DHF to form alternating sequences limits its ability to enable deconstruction at low loadings when employed in room temperature copolymerization reactions.

**Figure 1:**
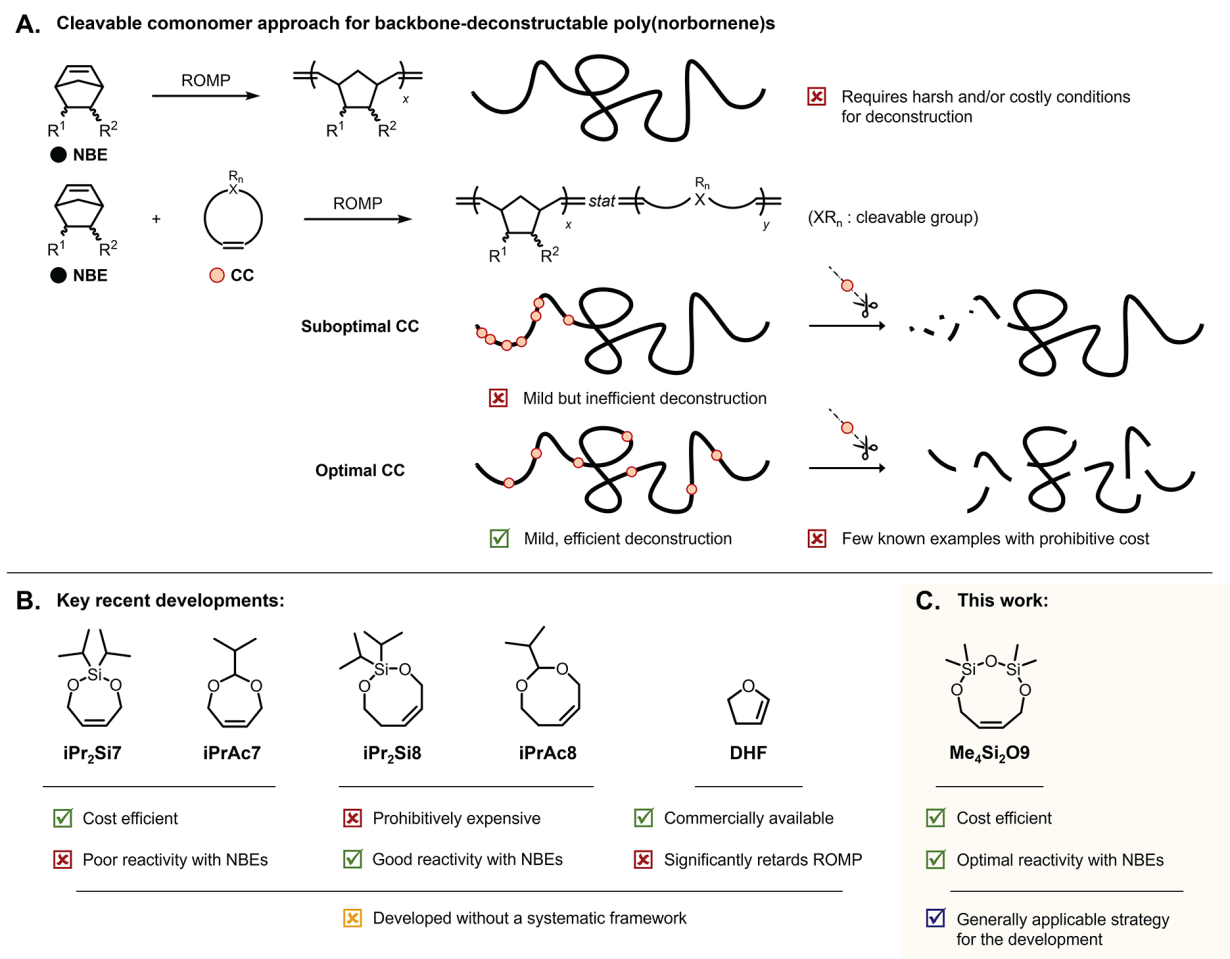
Contextualization for this study. (A) Cleavable comonomers (CCs) enable the deconstruction of poly­(norbornene)-based copolymers, with deconstruction efficiency dependent on copolymerization reactivity. (B) Existing CCs were developed without a systematic design framework and exhibit various limitations. (C) This work introduces a cost-efficient CC with optimal reactivity, developed through robust design criteria.

While the significance of designing optimal CCs has gained increasing recognition in recent years, there remain limited instances where a generalizable strategy has been successfully implemented in their development. In the context of ROMP, the often-reversible nature of CC incorporation introduces a large number of kinetic and thermodynamic variables that must be finely balanced within a narrow optimal range to achieve favorable reactivity. The simultaneous control of these parameters is often infeasible, and understanding of the most critical variables remains lacking. For example, parameters commonly correlated with ROMP *homopolymerization* reactivity (e.g., monomer ring strain, steric bulk, or HOMO energy levels)^53–56^ may not analogously correlate with CC incorporation in *copolymerizations*, further complicating the predictive discovery of improved CCs.

In this work, we address these challenges and present a predictive workflow that culminates in the discovery of a CC—**Me**_**4**_ **Si**_**2**_ **O9**—that exhibits excellent copolymerization reactivity across a broad range of NBEs (Figure 1C). We identify *equilibrium concentration* as a key parameter in the design of an optimal CC for ROMP, and use this insight into invent cyclic olefins with low equilibrium concentrations from readily available, inexpensive starting materials. This strategy is underpinned by the discovery that entropy—rather than ring strain—can serve as the dominant factor governing the copolymerization behavior of cyclic olefins. This insight challenges the prevailing belief that ROMP reactivity is governed by ring strain^57–60^ and provides a compelling explanation for how certain low-strain cyclic olefins can effectively copolymerize with high-strain monomers. Among these newly developed CCs, **Me**_**4**_ **Si**_**2**_ **O9** outperforms the current state-of-the-art, **iPr**_**2**_ **Si8**. Moreover, based on the prices of starting materials from common chemical vendors, **Me**_**4**_ **Si**_**2**_ **O9** is estimated to be about 30 times cheaper than **iPr**_**2**_ **Si8**, underscoring its potential for practical applications, with further cost reductions anticipated through process optimization or alternative synthetic routes.

## RESULTS AND DISCUSSION

### Stochastic Simulations Guide the Design of CCs

Our workflow for CC discovery begins with stochastic simulations of copolymerization processes to identify optimal reactivity parameters, which we define as the set of parameters that, after polymer deconstruction, gives oligomeric fragments with the largest decrease in weight-average molar mass relative to the starting polymer: *M*_w,frag_/*M*_w,pol_. Complete analysis of the copolymerization of CCs with NBEs is described using the model illustrated in Figure 2A, which involves four parameters, ***r*_M_**, ***r*_CC_**, **β**, and **γ**.^61^ This model can be simplified to three parameters—*r*_**M**_, *r*_**CC**_, and **β**—by assuming that the thermodynamics of propagation are unaffected by the penultimate unit, i.e., **β** = **γ**. Simulations were conducted using 5 mol % CC, a total DP of 500, and an initial total monomer concentration of 0.2 M.

**Figure 2:**
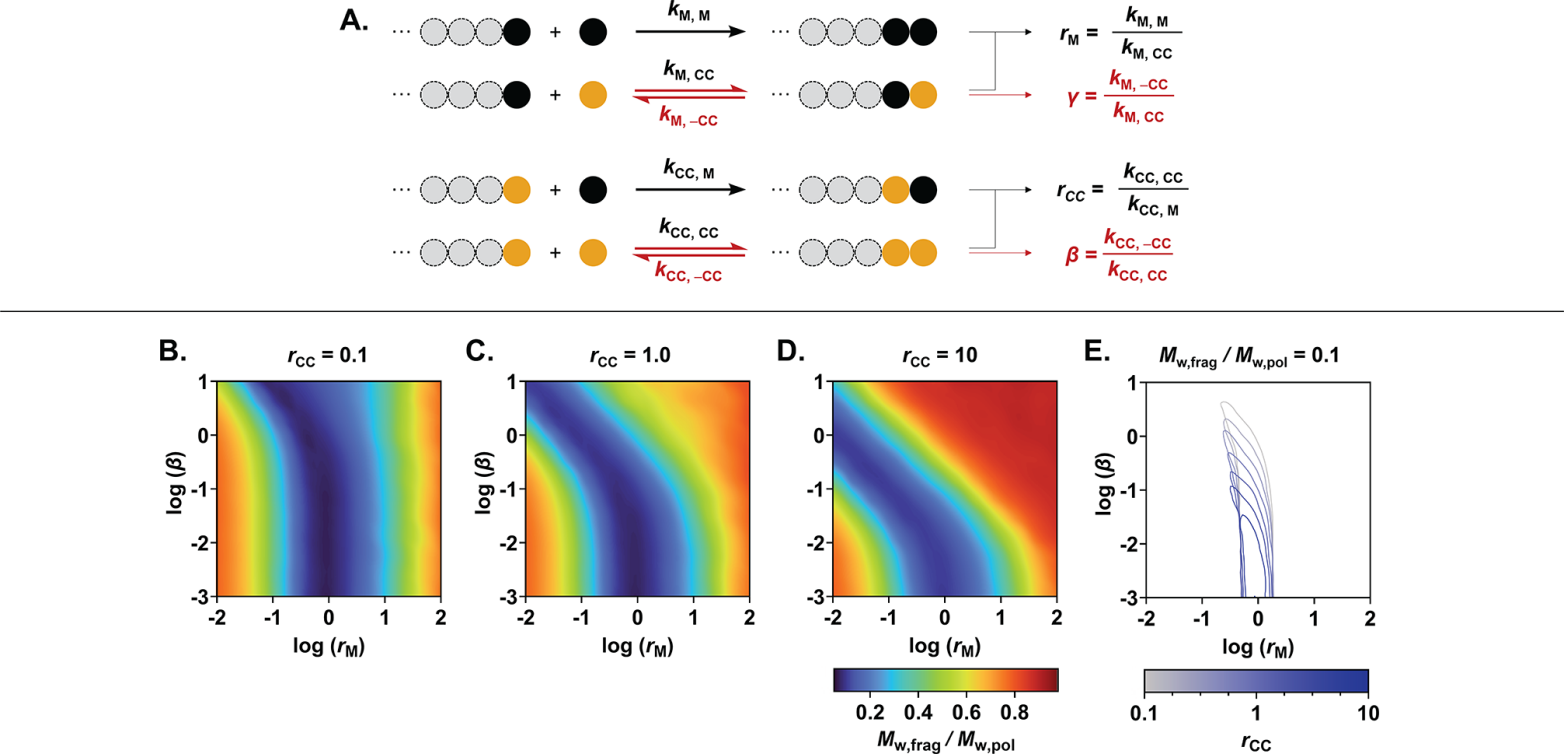
Stochastic simulations for the evaluation of the dependence of CC efficiency on copolymerization parameters. (A) Schematic of the model applied to the copolymerization of NBEs and cyclic olefin CCs. The simulations assumed a 5 mol % CC loading, a DP of 500, and an initial monomer concentration of 0.2 M for (B) ***r*_CC_** = 0.1, (C) ***r*_CC_** = 1.0, and (D) ***r*_CC_** = 10. The colors reflect the size of the deconstructed fragments relative to the original polymer size. (E) The contours for *M*_w,frag_/*M*_w,pol_ = 0.1 were compiled and visualized in a single plot for various ***r*_CC_** scenarios.

Figures 2B–D present heat maps of *M*_w,frag_/*M*_w,pol_ as a function of ***r*_M_** and **β** for three ***r*_CC_** values, where blue regions correspond to optimal ***r*_M_** and **β** pairs for a given ***r*_CC_**. Figure 2E displays contours where ***r*_M_**, **β**, and ***r*_CC_** give *M*_w,frag_/*M*_w,pol_ = 1/10, i.e., where *M*_w,frag_ is 10% that of *M*_w,pol_. Notably, these contours possess an overlapping region, where the optimal ***r*_M_** and **β** values are independent of ***r*_CC_** (for reasonable ***r*_CC_** values): **β** ≤ 10^–2^ M and ***r*_M_** between 0.8 and 1.1. Thus, we targeted CCs with **β** ≤ 10^–2^ M to confine uncertainty in new CC performance to a single experimentally measurable variable: ***r*_M_**.

Real systems may not strictly adhere to the assumption that **β** = **γ**; however, these parameters are interrelated through their mutual dependence on the Gibbs free energy of ROMP propagation (ΔG_p_), which guided our strategy to minimize ΔG_p_. We anticipated that a low ΔG_p_ would confer the advantage of reducing **β** and **γ** regardless of the substrate, promoting favorable copolymerization reactivity across a diverse range of monomers and enhancing the overall versatility of the CC. Throughout this work, we employed **β** as a measurable variable to probe the ΔG_p_ of the comonomers.

### Design of CCs with Low ROMP Equilibrium-Concentrations

As a starting point, the **β** of known CCs **iPr**_**2**_ **Si7** and **iPr**_**2**_ **Si8** were measured in chloroform at room temperature using an initial concentration of 200 mM, 1 mol % Grubbs third-generation catalyst (G3, structure shown in the Supporting Information (SI)), and 3 mol % benzoquinone (BQ), the latter of which inhibits olefin isomerization.^61^ The decrease in monomer concentration was monitored by real-time quantitative NMR (qNMR) until a plateau was reached (Figure 3A). The equilibrium concentrations for **iPr**_**2**_ **Si7** and **iPr**_**2**_ **Si8** measured under these conditions were 142 ± 1 mM and 49 ± 1 mM, respectively, which are consistent with the reported superior ability of **iPr**_**2**_ **Si8** to undergo homopolymerization compared to **iPr**_**2**_ **Si7**.^62^ We note that all equilibrium concentrations reported in this work are based on the assumption of negligible cyclic oligomer formation (see SI for further discussion).

**Figure 3:**
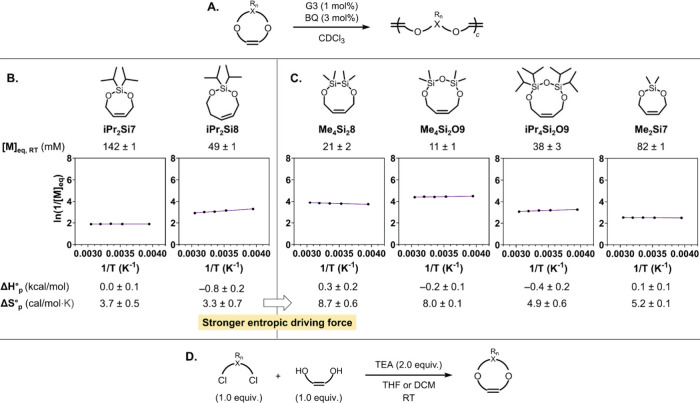
Synthesis and evaluation of the thermodynamic properties of the comonomers. (A) Equilibrium concentrations were measured from homopolymerization experiments. Van’t Hoff analyses were conducted for (B) existing and (C) new comonomers to determine ΔH°_p_ and ΔS°_p_. (D) The comonomers were synthesized in a single step from commercially available starting materials. We note that the comonomers were named based on three sequential features: (1) the identity and number of substituents on the Si center(s); (2) the number of Si and O atoms derived from the Si precursor; and (3) the ring size. For example, **Me**_**4**_ **Si**_**2**_ **O9** denotes a nine-membered cyclic comonomer bearing four methyl groups on two Si atoms, with two Si and one O atom derived from the Si precursor, dichlorotetramethyldisiloxane.

Following a similar protocol, equilibrium concentrations were measured across a temperature range of −20 to 55 °C. Subsequent Van’t Hoff analysis revealed ΔH°_p_ = 0.0 ± 0.1 kcal/mol and ΔS°_p_ = 3.7 ± 0.5 cal/mol·K for **iPr**_**2**_ **Si7**, and ΔH°_p_ = −0.8 ± 0.2 kcal/mol and ΔS°_p_ = 3.3 ± 0.7 cal/mol·K for **iPr**_**2**_ **Si8** (Figure 3B). Thus, while the enthalpic driving force for **iPr**_**2**_ **Si8** was 0.8 kcal/mol greater, aligning with the expected ring strain trend of cycloalkanes, both polymerizations were predominantly entropy-driven.^63,64^ This entropic driving force is attributed to the bifunctional silyl ether group, whose inherent flexibility creates significant increases in rotational and vibrational freedom upon transitioning from the ring-closed (monomer) to ring-opened (polymer) states,^62^ an effect analogous to that observed in ring-opening polymerizations of cyclic siloxanes.^65,66^ Building on these findings, we hypothesized that the key to optimal CC design would lie in further increasing ΔS°_p_ by introducing a greater number of flexible Si-based bonds, or by reducing the steric bulk in the vicinity of these flexible bonds to maximize the rotational and vibrational freedom after ring opening. Based on this hypothesis, we designed four monomers—**Me**_**4**_ **Si**_**2**_ **8**, **Me**_**4**_ **Si**_**2**_ **O9**, **iPr**_**4**_ **Si**_**2**_ **O9**, and **Me**_**2**_ **Si7**. In particular, the design of the siloxane-based comonomers **Me**_**4**_ **Si**_**2**_ **O9**, **iPr**_**4**_ **Si**_**2**_ **O9** was inspired by seminal studies from the 1960s, which revealed the exceptional conformational flexibility of Si–O–Si bonds, characterized by low bending and torsional energy barriers on the order of tenths of a kcal/mol.^67–69^ We also considered synthetic accessibility and cost as key criteria in monomer design. Each of the monomers can be synthesized from (Z)-but-2-ene-1,4-diol—one of the most affordable olefin-containing diols—and readily available Si-based building blocks (Figure 3C). For example, based on the price of starting materials from readily accessible chemical vendors, the approximate production cost of **Me**_**4**_ **Si**_**2**_ **O9** is 30 times lower than that of **iPr**_**2**_ **Si8** (see SI for detailed cost analysis). We anticipate that this cost could be further reduced through process optimization and/or alternative synthetic approaches, e.g., dehydrogenative coupling.

### Synthesis of CCs and Evaluation of ROMP Thermodynamics

**Me**_**4**_ **Si**_**2**_ **8**, **Me**_**4**_ **Si**_**2**_ **O9**, **iPr**_**4**_ **Si**_**2**_ **O9**, and **Me**_**2**_ **Si7** were synthesized in a single step from (*Z*)-but-2-ene-1,4-diol and a commercially available dichloro disilane, dichloro disiloxanes, or dichloro silane at room temperature, with yields of 71%, 75%, 69%, and 69%, respectively, requiring only simple vacuum distillation for purification (Figure 3D). The syntheses of **Me**_**4**_ **Si**_**2**_ **8** and **Me**_**4**_ **Si**_**2**_ **O9** were carried out on a decagram scale, yielding 17.2 and 18.1 g, respectively, from single batches.

Following the same protocol described above, equilibrium concentrations for **Me**_**4**_ **Si**_**2**_ **8**, **Me**_**4**_ **Si**_**2**_ **O9**, and **iPr**_**4**_ **Si**_**2**_ **O9** were measured by qNMR to be 21 ± 2 mM, 11 ± 1 mM, and 38 ± 3 mM, respectively, all of which were lower than that observed for **iPr**_**2**_ **Si8** (49 ± 1 mM) and further within the range of optimal performance as identified by stochastic simulations (Figure 3D). In support of our hypothesis regarding the impact of Si–based bonds on CC reactivity (*vide supra*), Van’t Hoff analysis revealed the ΔS°_p_ values increased substantially compared to those for **iPr**_**2**_ **Si7** and **iPr**_**2**_ **Si8**, with values of 8.7 ± 0.6, 8.0 ± 0.1, and 4.9 ± 0.6 cal/mol·K for **Me**_**4**_ **Si**_**2**_ **8**, **Me**_**4**_ **Si**_**2**_ **O9**, and **iPr**_**4**_ **Si**_**2**_ **O9**, respectively (Figure 3C). **Me**_**2**_ **Si7** showed an equilibrium concentration higher than **iPr**_**2**_ **Si8**, but lower than its isopropyl counterpart, **iPr**_**2**_ **Si7**, which was attributed to a stronger entropic driving force (ΔS°_p_ = 5.2 ± 0.1 cal/mol·K) from lowered steric bulk around the flexible Si-based bonds.

### Evaluation of the Copolymerization Behaviors of CCs with NBEs

The copolymerization reactivity of each CC described above with an *endo,exo*-norbornene diester (*endo,exo*-NBdE), **NB1**, was evaluated. **NB1** was selected for its reactivity being nearly equidistant between the *exo,exo* and *endo,endo* extremes on a logarithmic scale of ROMP propagation rates.^70^

For the accurate determination of copolymerization parameters, we applied a new workflow that relies on two data sets obtained under different copolymerization conditions (see SI for the detailed procedure and the evaluation of the reliability of this method). To generate the first data set, a 1:3 mixture of G3 and BQ was added to a 0.2 M 1:1 mixture of **NB1** and the CC, yielding a total monomer-to-G3 ratio of 500:1 (Figure 4A). For the second data set, the same G3/BQ mixture was added to a 0.2 M 2:1 mixture of **NB1** and the CC, maintaining the total monomer-to-G3 ratio at 500:1 (See SI for detailed procedures). **NB1** and CC conversions were measured by qNMR, and the two data sets were simultaneously fitted to the Izu-Lundberg equations to minimize the combined residual norm, thereby extracting kinetic and thermodynamic parameters (Figures 4B and S4).^61,71^ The homopropagation equilibrium concentration **β** values were preset to match those measured experimentally above; three-component fitting was performed to determine the reactivity ratios ***r*_M_** and ***r*_CC_**, as well as the cross-propagation equilibrium concentration **γ**.

**Figure 4:**
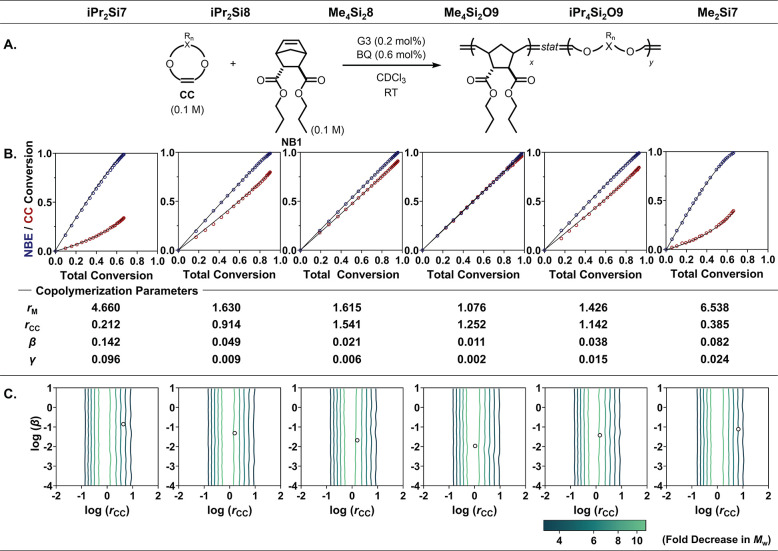
Evaluation of the reactivity of the comonomers. (A) Copolymerizations were conducted with an NBE of intermediate reactivity. (B) Monomer conversions were measured throughout the copolymerization and fitted to the Izu-Lundberg equation to determine copolymerization parameters. (C) Stochastic simulations predicted deconstruction efficiency based on these copolymerization parameters.

A general trend was observed where **β** scaled with **γ**, highlighting the effect of ΔG_p_ control on both parameters. Seven-membered ring CCs, **iPr**_**2**_ **Si7** and **Me**_**2**_ **Si7**, with their notably large ***r*_M_**, small ***r*_CC_**, and large **β** and **γ**, displayed a strongly gradient copolymerization behavior. By contrast, **iPr**_**2**_ **Si8**, **Me**_**4**_ **Si**_**2**_ **8**, **Me**_**4**_ **Si**_**2**_ **O9**, and **iPr**_**4**_ **Si**_**2**_ **O9** exhibited ***r*_M_** and ***r*_CC_** values closer to 1, with relatively small **β** and **γ**. Among these, **Me**_**4**_ **Si**_**2**_ **O9** copolymerization was closest to random, with ***r*_M_** nearest to 1 and the smallest **β** and **γ**. The almost identical conversion of **NB1** and **Me**_**4**_ **Si**_**2**_ **O9** throughout the copolymerization, coupled with its highly random behavior, suggests that **Me**_**4**_ **Si**_**2**_ **O9** may be ideal as a CC under these conditions, achieving an even distribution of cleavable bonds throughout the polymer backbone.

Using these measured copolymerization parameters as inputs, stochastic simulations were conducted to predict the deconstruction efficiencies (*M*_w,frag_/*M*_w,pol_) for each CC using 5 mol % CC loadings, total DPs of 500, and initial concentrations of 0.2 M (Figure 4C). Contours representing different deconstruction efficiencies are displayed, where the darkest shade indicates the least efficient (a 3.3-fold decrease in *M*_w_ upon deconstruction) and the lightest shade indicates the most efficient (a 11-fold decrease), with 8.2-, 6.1-, and 4.5-fold decreases shown in between (equidistant in log scale). The experimentally measured ***r*_M_** and **β** pair for each CC is marked as a circle on the plots, with its position relative to the contours indicating the theoretical deconstruction efficiency for each CC. The simulations predict that **iPr**_**2**_ **Si7** and **Me**_**2**_ **Si7** enable between 3.3- and 4.5-fold *M*_w_ decrease, while **iPr**_**2**_ **Si8**, **Me**_**4**_ **Si**_**2**_ **8**, and **iPr**_**4**_ **Si**_**2**_ **O9** enable close to 8.2- fold decreases. **Me**_**4**_ **Si**_**2**_ **O9** is within the 11-fold contour, suggesting that it exhibits the best performance.

Copolymers of **NB1** and each CC—**p­(NB1-** *co* **-CC)**s**—**were synthesized using 5 mol % CC loading, 0.2 mol % G3, and 0.6 mol % BQ at an initial monomer concentration of 0.2 M (theoretical DP = 500, Figure 5A). The resulting copolymers were first evaluated for their deconstruction rates. Prior studies have shown that substituents on silicon strongly influence the deconstruction kinetics of Si–O-based functional groups.^21^ Consistent with these findings, CCs bearing smaller substituents (e.g., Me) underwent cleavage substantially faster than those with larger substituents (e.g., iPr), following the general stability order: **Me**_**4**_ **Si**_**2**_ **8** < **Me**_**2**_ **Si7** ≈ **Me**_**4**_ **Si**_**2**_ **O9** < **iPr**_**2**_ **Si7** ≈ **iPr**_**4**_ **Si**_**2**_ **O9** (Figure S12). To compare the size of deconstruction fragments, the copolymers were subjected to alcoholysis using 2 M HCl in a mixture of *n*-PrOH and 1,4-dioxane at 50 °C for 6 h to induce complete Si–O bond cleavage. Size-exclusion chromatography (SEC) was used to measure the weight-average molar masses (*M*_w,SEC_) of the copolymers and their cleavage fragments (THF eluent; molar masses estimated relative to polystyrene standards; entries 2–7, Figure 5B, Figure S13). The SEC-measured trend in deconstruction efficiency matched the predictions of stochastic simulations in each case: **Me**_**2**_ **Si7** < **iPr**_**2**_ **Si7** ≪ **iPr**_**2**_ **Si8**, **Me**_**4**_ **Si**_**2**_ **8**, **iPr**_**4**_ **Si**_**2**_ **O9** < **Me**_**4**_ **Si**_**2**_ **O9**, with the latter displaying a 14-fold decrease in *M*_w,SEC_. To confirm complete bond cleavage, the deconstruction reaction duration was doubled, after which the fragments were analyzed by SEC, showing no further decrease in molar mass compared to the original conditions (Figure S14). We note that deconstruction under milder conditions (0.2 M HCl in a mixture of THF and water at room temperature) gave similar results (Figure S15A), as did a copolymer prepared under the same ROMP conditions but in the absence of BQ (**p­(NB1-** *co* **-Me**_**4**_ **Si**_**2**_ **O9)’**; entry 8, Figure 5B, Figure S16). Finally, a control homopolymer **pNB1** did not exhibit any molar mass decrease under the same deconstruction conditions, confirming the essential role of CCs (entry 1, Figure 5B, Figures S15B and S17).

**Figure 5:**
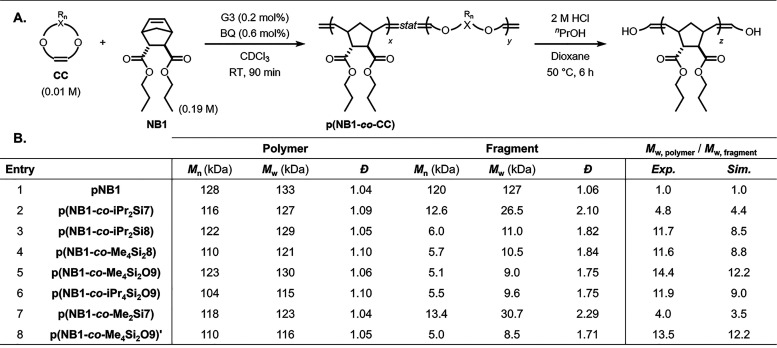
Evaluation of the performance of the comonomers. (A) Copolymers were prepared, deconstructed, and (B) analyzed using SEC with THF as the eluent. Molar masses were referenced against polystyrene standards. Simulated *M*_w,polymer_/*M*_w,fragment_ values were derived from stochastic simulations based on the measured copolymerization parameters; refer to Figures 4B and C.

### Applicability of **Me_4_Si_2_O9** to a Broad Spectrum of NBEs

Next, the applicability of **Me**_**4**_ **Si**_**2**_ **O9** to NBEs with varied ROMP reactivities was investigated. Two norbornene diimides (NBdIs)—*exo*-NBdI **NB2** and *endo*-NBdI **NB3**—were selected based on their reported fast and slow ROMP propagation kinetics, respectively.^70^ Copolymers **p­(NB2-** *co* **-Me**_**4**_ **Si**_**2**_ **O9)** and **p­(NB3-** *co* **-Me**_**4**_ **Si**_**2**_ **O9)**, each with 5 mol % CC loading, were prepared using 0.2 mol % G3 at an initial monomer concentration of 0.2 M (Figure 6A). Deconstruction of these copolymers with 0.2 M HCl resulted in a 9-fold decrease in *M*_w_ for **p­(NB2-** *co* **-Me**_**4**_ **Si**_**2**_ **O9)** and a 13-fold decrease for **p­(NB3-** *co* **-Me**_**4**_ **Si**_**2**_ **O9)**, demonstrating the excellent performance of **Me**_**4**_ **Si**_**2**_ **O9** across a spectrum of NBEs (Figures 6B and D). The control polymers, **pNB2** and **pNB3**, synthesized without **Me**_**4**_ **Si**_**2**_ **O9**, showed no molar mass decrease under the same deconstruction conditions (Figure S18).

**Figure 6:**
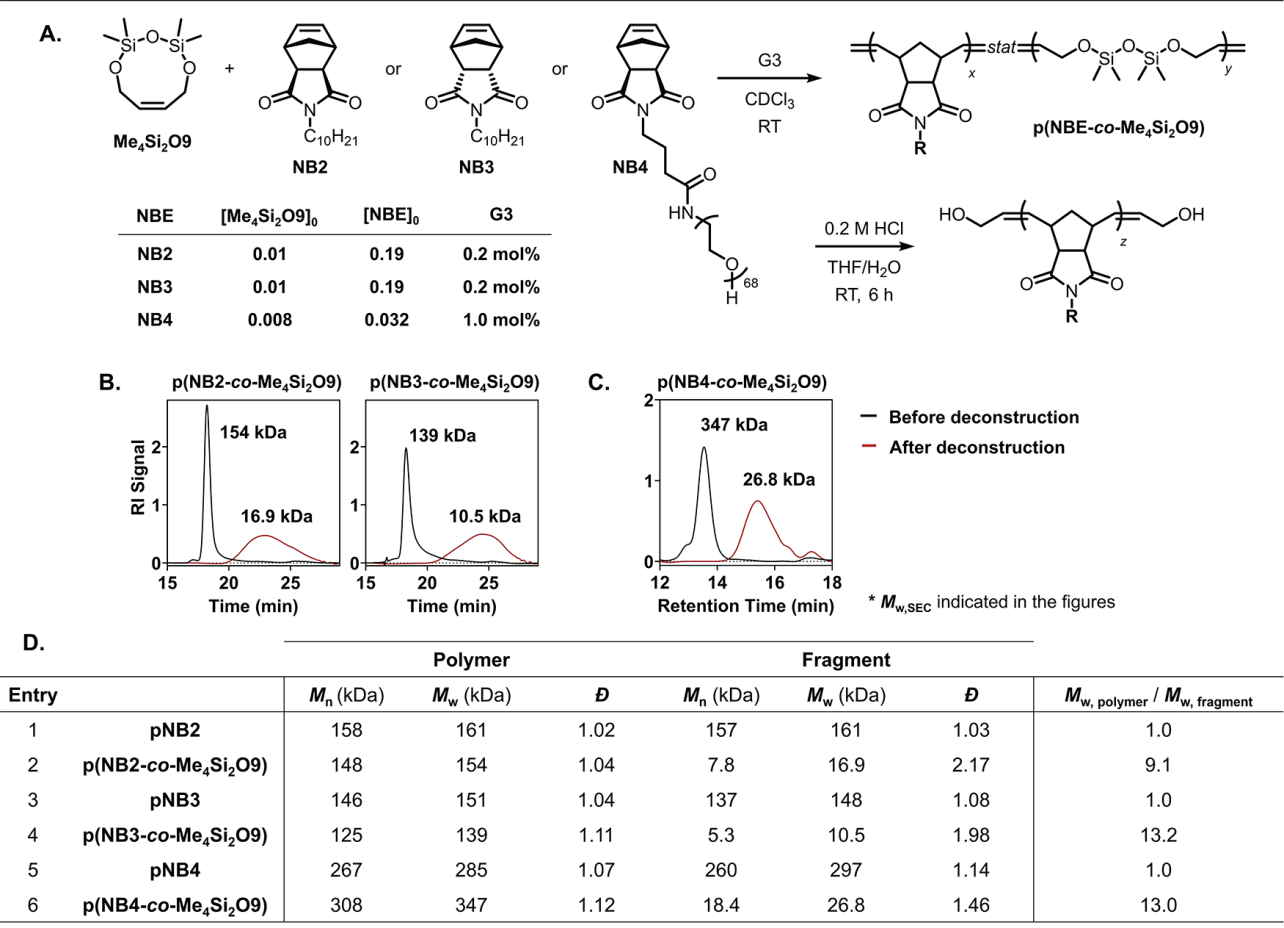
(A) The applicability of **Me**_**4**_ **Si**_**2**_ **O9** was evaluated using NBEs exhibiting both fast and slow ROMP kinetics, as well as in the synthesis of grafted polymers. Copolymers were prepared, deconstructed, and analyzed using SEC. (B) Copolymers incorporating **NB2** or **NB3** were analyzed using THF as the eluent, with molar masses referenced against polystyrene standards. (C) Copolymers incorporating **NB4** were analyzed using DMF as the eluent, and absolute molar masses were determined via SEC-MALS. Detailed values, including those for the control polymers, are provided in (D).

A known limitation of low-strain cyclic olefin CCs with moderate to high equilibrium concentrations is their inability to achieve efficient copolymerization in low-concentration polymerizations due to favorable reverse-propagation. This challenge becomes even more pronounced when the CC is incorporated as a minor component, resulting in a low absolute concentration. ROMP at low concentrations is common in the polymerization of macromonomers for constructing polymers with complex architectures. Here, we demonstrate that the low equilibrium concentrations of **Me**_**4**_ **Si**_**2**_ **O9** enables its effective use under such conditions. Graft polymers were synthesized from a polyethylene glycol (PEG)-macromonomer (**NB4**) and **Me**_**4**_ **Si**_**2**_ **O9** using 20 mol % CC loading and 1 mol % G3 at a total initial monomer concentration of 40 mM (the theoretical DP for **NB4** = 80), yielding **p­(NB4-** *co* **-Me**_**4**_ **Si**_**2**_ **O9)** (Figure 6A). A control polymer, **pNB4**, was synthesized using **NB4** and 1.25 mol % G3 at an initial concentration of 32 mM to match the theoretical DP and concentration of **NB4**. Subsequent acid hydrolysis using 0.2 M HCl and analysis via SEC-multiangle light scattering (SEC-MALS, DMF eluent) revealed that while **pNB4** exhibited no molar mass reduction (Figure S19), **p­(NB4-** *co* **-Me**_**4**_ **Si**_**2**_ **O9)** underwent a 13-fold decrease in *M*_w_ (Figures 6C and D). These results demonstrate the applicability of **Me**_**4**_ **Si**_**2**_ **O9** across a range of ROMP scenarios.

Finally, we note that the utility of **Me**_**4**_ **Si**_**2**_ **O9** as a CC for ROMP-derived thermosets, where it enables retention of key mechanical properties and thermal stability while delivering thermomechanical performance comparable to that of previously reported CCs, was recently reported.^72^

## Conclusion

In summary, we have successfully developed the first CC for ROMP that meets several essential criteria for practical applications—optimal reactivity and cost-effectiveness. This breakthrough was enabled by an improved understanding of the fundamental factors governing CC reactivity and formulating a broadly applicable design strategy for CC development that can be adapted to a range of chain-growth polymerizations with reversible propagation in the future. The effectiveness of the newly discovered CC **Me**_**4**_ **Si**_**2**_ **O9** was demonstrated in terms of both reactivity and cost, facilitating the synthesis of deconstructable ROMP-derived polymers for diverse applications.

## Supplementary Material



## Notes

Materials and analytical methods employed, detailed simulation and experimental procedures, cost estimates, and additional characterization data (PDF)

## References

[1] Kamaly N., Yameen B., Wu J., Farokhzad O. C. (2016). Degradable
Controlled-Release Polymers and Polymeric Nanoparticles: Mechanisms
of Controlling Drug Release. Chem. Rev..

[2] Rai R., Mantione D. (2023). The Future of Electronic
Materials Is···
Degradable!. J. Mater. Chem. C.

[3] Zhou J., Hsu T.-G., Wang J. (2023). Mechanochemical
Degradation and Recycling
of Synthetic Polymers. Angew. Chem., Int. Ed..

[4] Shi C., Quinn E. C., Diment W. T., Chen E. Y.-X. (2024). Recyclable and
(Bio)­degradable Polyesters in a Circular Plastics Economy. Chem. Rev..

[5] Kumar R., Sadeghi K., Jang J., Seo J. (2023). Mechanical, Chemical,
and Bio-recycling of Biodegradable Plastics: A Review. Sci. Total Environ..

[6] Čamdžić L., Haynes C. A., Stache E. E. (2024). An Old Polymer Class Revisited: Versatile,
Degradable, Non-Alternating Polyketones. Chem..

[7] Sazali N., Ibrahim H., Jamaludin A. S., Mohamed M. A., Salleh W. N. W., Abidin M. N. Z. (2020). A Short Review
on Polymeric Materials Concerning Degradable
Polymers. IOP Conf. Ser.: Mater. Sci. Eng..

[8] Aarsen C. V., Liguori A., Mattsson R., Sipponen M. H., Hakkarainen M. (2024). Designed to
Degrade: Tailoring Polyesters for Circularity. Chem. Rev..

[9] Haider T. P., Voelker C., Kramm J., Landfester K., Wurm F. R. (2019). Plastics of the Future? The Impact of Biodegradable
Polymers on the Environment and on Society. Angew. Chem., Int. Ed..

[10] Broda M., Yelle D. J., Serwańska-Leja K. (2024). Biodegradable Polymers
in Veterinary Medicine—A Review. Molecules.

[11] Shieh P., Zhang W., Husted K. E. L., Kristufek S. L., Xiong B., Lundberg D. J., Lem J., Veysset D., Sun Y., Nelson K. A., Plata D. L., Johnson J. A. (2020). Cleavable Comonomers
Enable Degradable, Recyclable Thermoset Plastics. Nature.

[12] Davydovich O., Paul J. E., Feist J. D., Aw J. E., Balta
Bonner F. J., Lessard J. J., Tawfick S., Xia Y., Sottos N. R., Moore J. S. (2022). Frontal Polymerization of Dihydrofuran
Comonomer Facilitates Thermoset Deconstruction. Chem. Mater..

[13] Lloyd E. M., Cooper J. C., Shieh P., Ivanoff D. G., Parikh N. A., Mejia E. B., Husted K. E. L., Costa L. C., Sottos N. R., Johnson J. A., Moore J. S. (2023). Efficient Manufacture,
Deconstruction,
and Upcycling of High-Performance Thermosets and Composites. ACS Appl. Eng. Mater..

[14] Kiel G. R., Lundberg D. J., Prince E., Husted K. E. L., Johnson A. M., Lensch V., Li S., Shieh P., Johnson J. A. (2022). Cleavable
Comonomers for Chemically Recyclable Polystyrene: A General Approach
to Vinyl Polymer Circularity. J. Am. Chem. Soc..

[15] Ko K., Lundberg D. J., Johnson A. M., Johnson J. A. (2024). Mechanism-Guided
Discovery of Cleavable Comonomers for Backbone Deconstructable Poly­(methyl
methacrylate). J. Am. Chem. Soc..

[16] Luzel B., Gil N., Désirée P., Monot J., Bourissou D., Siri D., Gigmes D., Martin-Vaca B., Lefay C., Guillaneuf Y. (2023). Development of an Efficient Thionolactone
for Radical Ring-Opening Polymerization by a Combined Theoretical/Experimental
Approach. J. Am. Chem. Soc..

[17] Elliss H., Dawson F., Nisa Q. U., Bingham N. M., Roth P. J., Kopeć M. (2022). Fully Degradable
Polyacrylate Networks from Conventional
Radical Polymerization Enabled by Thionolactone Addition. Macromolecules.

[18] Lundberg D. J., Ko K., Kilgallon L. J., Johnson J. A. (2024). Defining Reactivity–Deconstructability
Relationships for Copolymerizations Involving Cleavable Comonomer
Additives. ACS Macro Lett..

[19] Lefay C., Guillaneuf Y. (2023). Recyclable/Degradable
Materials via the Insertion of
Labile/Cleavable Bonds Using a Comonomer Approach. Prog. Polym. Sci..

[20] Morris P. T., Watanabe K., Albanese K. R., Kent G. T., Gupta R., Gerst M., Read de
Alaniz J., Hawker C. J., Bates C. M. (2024). Scalable
Synthesis of Degradable Copolymers Containing α-Lipoic Acid
via Miniemulsion Polymerization. J. Am. Chem.
Soc..

[21] Shieh P., Nguyen H. V.-T., Johnson J. A. (2019). Tailored Silyl Ether Monomers Enable
Backbone-Degradable Polynorbornene-Based Linear, Bottlebrush, and
Star Copolymers through ROMP. Nat. Chem..

[22] Calderón-Díaz A., Boggiano A. C., Xiong W., Kaiser N., Gutekunst W. R. (2024). Degradable
N-Vinyl Copolymers through Radical Ring-Opening Polymerization of
Cyclic Thionocarbamates. ACS Macro Lett..

[23] Grubbs, R. H. ; Khosravi, E. Handbook of Metathesis, 2nd ed.; Wiley-VCH: 2015; Vol. 3.

[24] Blosch S.
E., Scannelli S. J., Alaboalirat M., Matson J. B. (2022). Complex Polymer
Architectures Using Ring-Opening Metathesis Polymerization: Synthesis,
Applications, and Practical Considerations. Macromolecules.

[25] Sutthasupa S., Shiotsuki M., Sanda F. (2010). Recent Advances in Ring-Opening Metathesis
Polymerization, and Application to Synthesis of Functional Materials. Polym. J..

[26] Hou W., Yin X., Zhou Y., Shi Y., Chen Y. (2024). Ring-Opening Metathesis
Polymerization for Synthesizing Molecular Bottlebrushes via the Grafting-Through
Strategy. J. Polym. Sci..

[27] Chang A. B., Miyake G. M., Grubbs R. H. (2014). Sequence-Controlled
Polymers by Ruthenium-Mediated
Ring-Opening Metathesis Polymerization. ACS
Symp. Ser..

[28] Zhou C., Hou C., Chen W., Wang L., Cheng J. (2022). Progress of Application
of Ring-Opening Metathesis Polymerization (ROMP) in the Synthesis
of Star Polymers. Acta Chim. Sin..

[29] Johnson J. A., Lu Y. Y., Burts A. O., Xia Y., Durrell A. C., Tirrell D. A., Grubbs R. H. (2010). Drug-Loaded, Bivalent-Bottle-Brush
Polymers by Graft-Through ROMP. Macromolecules.

[30] Smith D., Pentzer E. B., Nguyen S. T. (2007). Bioactive
and Therapeutic ROMP Polymers. Polym. Rev..

[31] Gandra U. R., Podiyanachari S. K., Bazzi H. S., Al-Hashimi M. (2023). Recent Advances
in Drug Release, Sensing, and Cellular Uptake of Ring-Opening Metathesis
Polymerization (ROMP) Derived Poly­(olefins). ACS Omega.

[32] Jiang Q., Chen Q., Jiang F., Chen C., Verpoort F. (2018). A Review of
the Ring-Opening Metathesis Polymerization Involving Norbornene or
Its Derivatives. Mater. Rep..

[33] Kessler M. R., White S. R. (2002). Cure Kinetics of
the Ring-Opening Metathesis Polymerization
of Dicyclopentadiene. J. Polym. Sci. A Polym.
Chem..

[34] Robertson I. D., Yourdkhani M., Centellas P. J., Aw J. E., Ivanoff D. G., Goli E., Lloyd E. M., Dean L. M., Sottos N. R., Geubelle P. H., Moore J. S., White S. R. (2018). Rapid Energy-Efficient
Manufacturing of Polymers and Composites via Frontal Polymerization. Nature.

[35] Rule J. D., Moore J. S. (2002). ROMP Reactivity
of Endo- and Exo-Dicyclopentadiene. Macromolecules.

[36] Boadi F. O., Zhang J., Yu X., Bhatia S. R., Sampson N. S. (2020). Alternating
Ring-Opening Metathesis Polymerization Provides Easy Access to Functional
and Fully Degradable Polymers. Macromolecules.

[37] Debsharma T., Behrendt F. N., Laschewsky A., Schlaad H. (2019). Ring-Opening Metathesis
Polymerization of Biomass-Derived Levoglucosenol. Angew. Chem., Int. Ed..

[38] Elling B. R., Su J. K., Xia Y. (2020). Degradable
Polyacetals/Ketals from
Alternating Ring-Opening Metathesis Polymerization. ACS Macro Lett..

[39] Feist J. D., Lee D. C., Xia Y. (2022). A Versatile
Approach for the Synthesis
of Degradable Polymers via Controlled Ring-Opening Metathesis Copolymerization. Nat. Chem..

[40] Feist J. D., Xia Y. (2020). Enol Ethers Are Effective Monomers
for Ring-Opening Metathesis Polymerization:
Synthesis of Degradable and Depolymerizable Poly­(2,3-dihydrofuran). J. Am. Chem. Soc..

[41] Fishman J. M., Kiessling L. L. (2013). Synthesis
of Functionalizable and Degradable Polymers
by Ring-Opening Metathesis Polymerization. Angew.
Chem., Int. Ed..

[42] Moatsou D., Nagarkar A., Kilbinger A. F. M., O’Reilly R. K. (2016). Degradable
Precision Polynorbornenes via Ring-Opening Metathesis Polymerization. J. Polym. Sci., Part A: Polym. Chem..

[43] Liang Y., Sun H., Cao W., Thompson M. P., Gianneschi N. C. (2020). Degradable
Polyphosphoramidate via Ring-Opening Metathesis Polymerization. ACS Macro Lett..

[44] Haider T., Shyshov O., Suraeva O., Lieberwirth I., von Delius M., Wurm F. R. (2019). Long-Chain Polyorthoesters
as Degradable
Polyethylene Mimics. Macromolecules.

[45] Mallick A., Xu Y., Lin Y., He J., Chan-Park M. B., Liu X.-W. (2018). Oxadiazabicyclooctenone as a Versatile
Monomer for
the Construction of pH-Sensitive Functional Polymers via ROMP. Polym. Chem..

[46] Steinbach T., Alexandrino E. M., Wurm F. R. (2013). Unsaturated Poly­(phosphoester)­s via
Ring-Opening Metathesis Polymerization. Polym.
Chem..

[47] Chang C. C., Emrick T. (2014). Functional Polyolefins
Containing Disulfide and Phosphoester
Groups: Synthesis and Orthogonal Degradation. Macromolecules.

[48] Sun H., Liang Y., Thompson M. P., Gianneschi N. C. (2021). Degradable
Polymers via Olefin Metathesis Polymerization. Prog. Polym. Sci..

[49] Wang X., Wen Y., Wang Y., Li W., Lu X., You W. (2024). 7-Oxa-2,3-Diazanorbornene:
A One-Step Accessible Monomer for Living Ring-Opening Metathesis Polymerization
to Produce Backbone-Biodegradable Polymers. CCS Chem..

[50] Steinbach T., Alexandrino E. M., Wahlen C., Landfester K., Wurm F. R. (2014). Poly­(phosphonate)­s
via Olefin Metathesis: Adjusting
Hydrophobicity and Morphology. Macromolecules.

[51] Arrington K. J., Waugh J. B., Radzinski S. C., Matson J. B. (2017). Photo- and Biodegradable
Thermoplastic Elastomers: Combining Ketone-Containing Polybutadiene
with Polylactide Using Ring-Opening Polymerization and Ring-Opening
Metathesis Polymerization. Macromolecules.

[52] Huang B., Wei M., Vargo E., Qian Y., Xu T., Toste F. D. (2021). Backbone-Photodegradable
Polymers by Incorporating Acylsilane Monomers via Ring-Opening Metathesis
Polymerization. J. Am. Chem. Soc..

[53] Kilgallon L. J., McFadden T. P., Sigman M. S., Johnson J. B. (2024). Tricyclononenes
and Tricyclononadienes as Efficient Monomers for Controlled ROMP:
Understanding Structure–Propagation Rate Relationships and
Enabling Facile Post-Polymerization Modification. Chem. Sci..

[54] Scannelli S. J., Paripati A., Weaver J. R., Vu C., Alaboalirat M., Troya D., Matson J. B. (2023). Influence of the Norbornene Anchor
Group in Ru-Mediated Ring-Opening Metathesis Polymerization: Synthesis
of Linear Polymers. Macromolecules.

[55] Scannelli S. J., Alaboalirat M., Troya D., Matson J. B. (2023). Influence of the
Norbornene Anchor Group in Ru-Mediated Ring-Opening Metathesis Polymerization:
Synthesis of Bottlebrush Polymers. Macromolecules.

[56] Radzinski S. C., Foster J. C., Chapleski R. C., Troya D., Matson J. B. (2016). Bottlebrush Polymer Synthesis by Ring-Opening Metathesis
Polymerization: The Significance of the Anchor Group. J. Am. Chem. Soc..

[57] Hejl A., Scherman O. A., Grubbs R. H. (2005). Ring-Opening
Metathesis Polymerization
of Functionalized Low-Strain Monomers with Ruthenium-Based Catalysts. Macromolecules.

[58] Hlil A. R., Balogh J., Moncho S., Su H.-L., Tuba R., Brothers E. N., Al-Hashimi M., Bazzi H. S. (2017). Ring-Opening Metathesis
Polymerization (ROMP) of Five- to Eight-Membered Cyclic Olefins: Computational,
Thermodynamic, and Experimental Approach. J.
Polym. Sci. A Polym. Chem..

[59] Sylvester K. R., Zovinka J. R., Milrod M. L., Stubin A. K., Rojas-Merchan A., Alexander K., Elling B. R. (2025). Allylic Epoxides Increase the Strain
Energy of Cyclic Olefin Monomers for Ring-Opening Metathesis Polymerization. Angew. Chem., Int. Ed..

[60] Kobayashi, S. Ring-Opening Metathesis Polymerization. In Encyclopedia of Polymeric Nanomaterials; Kobayashi, S. ; Müllen, K. , Eds.; Springer: 2015. 10.1007/978-3-642-29648-2_200.

[61] Lundberg D. J., Kilgallon L. J., Cooper J. C., Starvaggi F., Xia Y., Johnson J. A. (2024). Accurate Determination of Reactivity Ratios for Copolymerization
Reactions with Reversible Propagation Mechanisms. Macromolecules.

[62] Johnson A. M., Husted K. E. L., Kilgallon L. J., Johnson J. A. (2022). Orthogonally Deconstructable
and Depolymerizable Polysilylethers via Entropy-Driven Ring-Opening
Metathesis Polymerization. Chem. Commun..

[63] Hodge P., Colquhoun H. M. (2005). Recent
Work on Entropically-Driven Ring-Opening Polymerizations:
Some Potential Applications. Polym. Adv. Technol..

[64] Marsella M. J., Maynard H. D., Grubbs R. H. (1997). Template-Directed Ring-Closing Metathesis:
Synthesis and Polymerization of Unsaturated Crown Ether Analogs. Angew. Chem., Int. Ed. Engl..

[65] Carmichael J. B., Winger R. (1965). Cyclic Distribution
in Dimethylsiloxanes. J. Polym. Sci., Part A.

[66] Suter U. W., Mutter M., Flory P. J. (1976). Macrocyclization
Equilibriums. 2.
Poly­(dimethylsiloxane). J. Am. Chem. Soc..

[67] Durig J. R., Flanagan M. J., Kalasinsky V. F. (1977). The Determination of the Potential
Function Governing the Low Frequency Bending Mode of Disiloxane. J. Chem. Phys..

[68] Scott D. W., Messerly J. F., Todd S. S., Guthrie G. B., Hossenlopp I. A., Moore R. T., Osborn A., Berg W. T., McCullough J. P. (1961). Hexamethyldisiloxane:
Chemical Thermodynamic Properties and Internal Rotation about the
Siloxane Linkage. J. Phys. Chem..

[69] Grigoras, S. ; Lane, T. H. Conformational Analysis of Substituted Polysiloxane Polymers. In Silicon-Based Polymer Science; American Chemical Society: 1990; Vol. 224, pp 125–144. 10.1021/ba-1990-0224.ch007.

[70] Chang A. B., Lin T.-P., Thompson N. B., Luo S.-X. L., Liberman-Martin A. L., Chen H.-Y., Lee B., Grubbs R. H. (2017). Design, Synthesis,
and Self-Assembly of Polymers with Tailored Graft Distributions. J. Am. Chem. Soc..

[71] Howell J. A., Izu M., O’Driscoll K. F. (1970). Copolymerization
with Depropagation.
III. Composition and Sequence Distribution from Probability Considerations. J. Polym. Sci. A1.

[72] Ko K., Mejia E. B., Fowler H. E., Nguyen S. T., AlFaraj Y., Wang Y., Leguizamon S. C., Sottos N. R., Johnson J. A. (2025). Multi-Generation
Recycling of Thermosets Enabled by Fragment Reactivation. J. Am. Chem. Soc..

