# Supplementary material for: Entropy Drives the Predictive Discovery of an Optimal Cleavable Comonomer for ROMP
Source: ACS Cent Sci. 2025 Jul 23;11(8):1408–16. doi: 10.1021/acscentsci.5c00521 (PMC12395298; doi:10.1021/acscentsci.5c00521)
Supplement: Supplementary file 1 [file oc5c00521_si_001.pdf]

Supporting Information for:

Entropy Drives the Predictive Discovery of an Optimal Cleavable Comonomer for ROMP

Kwangwook Ko,[1] Piper L. MacNicol,[1] Mingming Zhu,[2] Lei Zhang,[2] Saifudin M. Abubakar,[3] and Jeremiah A. Johnson[1]*

Affiliations

[1]Department of Chemistry, Massachusetts Institute of Technology, 77 Massachusetts Avenue, Cambridge, MA 02139, U.S.A.

[2]ExxonMobil Asia Pacific Research and Development Co., Ltd., Shanghai 200241, China

[3]ExxonMobil Asia Pacific Pte. Ltd., 1 HarbourFront Place, 098633, Singapore

*Correspondence to: jaj2109@mit.edu

Table of Contents

1. General Experimental Details……………………………………………S4

1.1. General Considerations……………………………………………S4

1.2. General Materials Information……………………………………S4

1.3. General Analytical Information…………………………………S5

2. Simulation Details………………………………………………………S6

2.1. Generation of Figures 2B to D……………………………………S6

2.2. Generation of Figure 2E…………………………………………S7

2.3. Generation of Figure 4C…………………………………………S7

3. Experimental Procedures………………………………………………S8

3.1. Preparation of Stock Solutions……………………………………S8

3.2. Equilibrium Concentration Measurement…………………………S8

3.3. Van't Hoff Analysis………………………………………………S8

3.4. Kinetic Experiments………………………………………………S9

3.5. Copolymer Synthesis……………………………………………S10

3.6. Copolymer Deconstruction………………………………………S11

3.7. Safety Statement…………………………………………………S12

4. Synthesis and Characterization of Monomers………………………S13

4.1. Synthesis of **Me_4Si_28**……………………………………………S13

4.2. Synthesis of **Me_4Si_2O9**…………………………………………S14

4.3. Synthesis of **iPr_4Si_2O9**…………………………………………S15

4.4. Synthesis of **Me_2Si7**……………………………………………S16

4.5. Safety Statement…………………………………………………S16

5. Fitting Details…………………………………………………………S17

5.1. Izu-Lundberg Equation……………………………………………S17

5.2. Method Development………………………………………………S17

5.3. Evaluation of the Method…………………………………………S18

5.4. Fitting Results……………………………………………………S19

6. Cost Estimation..S20

6.1. **Me_4Si_2O9**..S20

6.2. **iPr_2Si8**..S21

7. Supplementary Data and Discussions..S23

7.1. Equilibrium Concentration Measurement..S23

7.2. Van't Hoff Analysis..S24

7.3. Copolymer Deconstruction Studies..S29

8. NMR Spectra..S34

9. References and Notes..S37

1. General Experimental Details

1.1. General Considerations

All reactions were performed using standard Schlenk techniques unless stated otherwise. All glassware was dried in a 120 °C oven overnight or flame-dried prior to use. Molecular sieves (4Å) were activated by heating at 220 °C for three days under a nitrogen atmosphere, followed by an additional three days under vacuum before being stored in a glovebox.

1.2. General Materials Information

Unless otherwise noted, all reagents and starting materials were purchased from commercial vendors (Millipore-Sigma, Alfa Aesar, Strem, Ambeed, Beantown Chemical, Apollo Scientific, or Matrix Scientific) and used as received. Anhydrous tetrahydrofuran (THF), dichloromethane (DCM), *n*-propyl alcohol (^{n}PrOH) and triethylamine (TEA) were purchased from Millipore-Sigma packaged in Sure/Seal™ bottles and used as received. Deuterated chloroform ($CDCl_3$) was purchased from Cambridge Isotope Laboratories (CIL), dried over CaH_2, distilled, degassed by four freeze-pump-thaw cycles, and stored in a glovebox over activated 4Å molecular sieves.

Grubbs 2nd-generation catalyst (G2) was obtained from Materia (Grubbs Catalyst® C848) and used as received. Grubbs 3rd-generation catalyst (G3) was synthesized from G2 following established literature procedures.[1] **iPr$_2$Si7** and **iPr$_2$Si8** were synthesized according to the literature[2] and purified by vacuum distillation before use. Norbornene derivatives (NBEs) **NB1**,[3] **NB2**,[4] **NB3**,[5] and **NB4**[6] were synthesized based on reported methods.

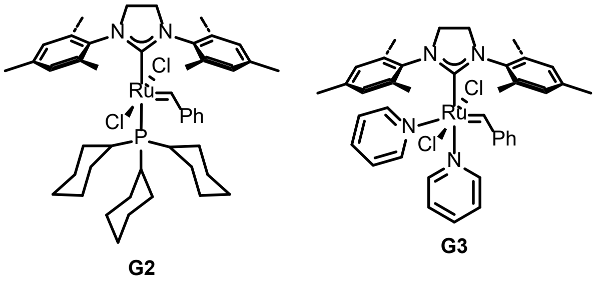

Figure S1. Chemical structures of Grubbs catalysts used for this work.

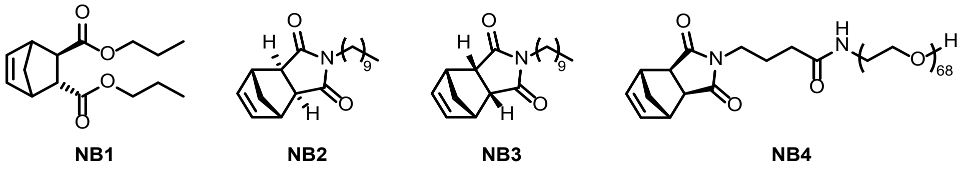

Figure S2. Chemical structures of NBEs used for this work.

1.3. General Analytical Information

NMR spectra were recorded on either Bruker AVANCE III DRX 400 or Neo 500 spectrometers at room temperature. Proton (^{1}H) chemical shifts are indicated in ppm and are calibrated to residual solvent peaks ($CDCl_3$: δ 7.26 ppm). All carbon (^{13}C) NMR recordings are proton-decoupled and their chemical shifts are also shown in ppm, referenced to the solvent's carbon resonance ($CDCl_3$: δ 77.16 ppm). All NMR data were analyzed and processed using MestReNOVA. Quantitative ^{1}H NMR spectroscopy were performed using 1,4-bis(trimethylsilyl)benzene as an internal standard.

High-resolution mass spectra were recorded on JEOL AccuTOF 4G equipped with an ionSense DART.

Analytical size exclusion chromatography (SEC) was performed in HPLC-grade THF using an Agilent 1260 Infinity system, equipped with an Agilent PLgel guard column (5 µm; 50 x 7.5 mm) and three Agilent PLgel analytical columns (5 µm; 300 x 7.5 mm; 105, 104, and 103 Å pore sizes). The analysis was conducted at 35 °C with a flow rate of 1.0 mL/min. The instrument was calibrated with polystyrene standards. Molecular weight and dispersity values were calculated using ChemStation GPC Data Analysis Software (rev. B.01.01), based on the refractive index signal.

SEC-multiangle light scattering (SEC-MALS) data were collected using an Agilent 1260 Infinity system with a Wyatt Optilab T-rEX differential refractive index detector, a Wyatt DAWN EOS 18-angle laser light scattering detector, and two Shodex KD-806M columns in tandem. Elution was carried out in N,N-dimethylformamide (DMF) containing 25 mM lithium bromide, at a flow rate of 1.0 mL/min at 60 °C.

2. Simulation Details

2.1. Generation of Figures 2B to D

Stochastic copolymerization simulations were conducted targeting a chain length of 500 monomers and simulating a total of 400 chains with an initial concentration set at 0.2 M. To generate the plots, 144 pairs of $\boldsymbol{r}_{\mathrm{M}}$ and $\boldsymbol{\beta}$ were simulated for a fixed $\boldsymbol{r}_{\mathrm{CC}}$. This included all combinations of 12 different $\boldsymbol{r}_{\mathrm{M}}$ values chosen at equal intervals in logspace (-2 to 2) and 12 different $\boldsymbol{\beta}$ values chosen at equal intervals in logspace (-3 to 1). $\boldsymbol{\gamma}$ was assumed to be the same as $\boldsymbol{\beta}$. The data were interpolated to create a gradient illustration.

For each combination of $\boldsymbol{r}_{\mathrm{M}}$ and $\boldsymbol{\beta}$, a stochastic simulation of the copolymerization process was performed. A polymer array was generated to represent the composition of each polymer chain, initialized with a monomer selected according to the initial monomer amounts. The type of reaction was randomly selected based on the cumulative propagation probabilities calculated from the current monomer concentrations, reactivity ratios, and equilibrium concentrations. Depending on the reaction, a polymer chain was randomly chosen for either propagation or reverse propagation. The selected chain was either extended by adding M or CC or shortened by removing CC. After each step, the length, molecular weight, and terminal monomer identity of the chain, as well as the remaining amount of monomers and their concentrations, were updated. The propagation probabilities were recalculated to reflect the new composition of the monomer pool concentrations and the current terminal monomer of each chain. The simulation continued until the predetermined number of monomers had reacted. For each polymer chain in the array, the runs of monomers (repeated elements), their lengths, and their values were analyzed using a predefined function. The weight-average molecular weight of the non-degradable segments was calculated based on the lengths of consecutive Ms.

Using the surfc function, a surface plot was generated to represent the decrease in polymer molecular weight after deconstruction across different combinations of reactivity ratios. For enhanced visualization, the axes were set to a logarithmic scale.

2.2. Generation of Figure 2E

Stochastic copolymerization simulations were conducted targeting a chain length of 500 monomers and simulating a total of 1,000 chains with an initial concentration set at 0.2 M. To generate the plots, 1,152 combinations of $\boldsymbol{r}_{\mathrm{M}}$, $\boldsymbol{r}_{\mathrm{CC}}$, and $\boldsymbol{\beta}$ were simulated. This included all combinations of 12 different $\boldsymbol{r}_{\mathrm{M}}$ values chosen at equal intervals in logspace (-2 to 2), eight different $\boldsymbol{r}_{\mathrm{CC}}$ values chosen at equal intervals in logspace (-1 to 0.75), and 12 different $\boldsymbol{\beta}$ values chosen at equal intervals in logspace (-3 to 1). The data were interpolated to create a gradient illustration.

For each combination of $\boldsymbol{r}_{\mathrm{M}}$, $\boldsymbol{r}_{\mathrm{CC}}$, and $\boldsymbol{\beta}$, a stochastic simulation of the copolymerization process was conducted as described in **2.1.** Contours were visualized for each $\boldsymbol{r}_{\mathrm{CC}}$ where the decrease in polymer molecular weight after deconstruction equaled 0.1 (a 10-fold decrease).

2.3. Generation of Figure 4C

Stochastic copolymerization simulations were conducted targeting a chain length of 500 monomers and simulating a total of 1,000 chains with an initial concentration set at 0.2 M. To generate the plots, $\boldsymbol{r}_{\mathrm{CC}}$ and $\boldsymbol{\gamma}$ values were preset to the experimentally determined values, and 144 combinations of $\boldsymbol{r}_{\mathrm{M}}$ and $\boldsymbol{\beta}$ were simulated. This covered all combinations of 12 different $\boldsymbol{r}_{\mathrm{M}}$ values chosen at equal intervals in logspace (-2 to 2) and 12 different $\boldsymbol{\beta}$ values chosen at equal intervals in logspace (-4 to 1). The data were interpolated to create a gradient illustration. For each combination of $\boldsymbol{r}_{\mathrm{M}}$ and $\boldsymbol{\beta}$, a stochastic simulation of the copolymerization process was conducted as described in 2.1. Contours were visualized at the levels of 0.09, 0.122, 0.164, 0.222, and 0.3.

3. Experimental Procedures

3.1. Preparation of Stock Solutions

A solution of 1,4-bis(trimethylsilyl)benzene in $CDCl_3$ was prepared by dissolving a known amount of the compound in $CDCl_3$ to achieve a concentration of approximately 10 mM. Stock solutions of **iPr$_2$Si7**, **iPr$_2$Si8**, **Me$_4$Si$_2$8**, **Me$_4$Si$_2$O9**, **iPr$_4$Si$_2$O9**, and **Me$_2$Si7** were prepared at a concentration of 200 mM in the 1,4-bis(trimethylsilyl)benzene/$CDCl_3$ solution. Likewise, stock solutions of **NB1**, **NB2**, and **NB3** were prepared at 200 mM in the same 1,4-bis(trimethylsilyl)benzene/$CDCl_3$ solution. All stock solutions were prepared freshly before use.

3.2. Equilibrium Concentration Measurement

600 µL of the CC stock solution was transferred to an NMR tube, sealed with a penetrable rubber septum and electrical tape, and removed from the glovebox. A G3 solution was prepared separately by dissolving 14.5 mg of G3 and 7.3 mg of benzoquinone in 500 µL of $CDCl_3$ (without 1,4-bis(trimethylsilyl)benzene). The G3 solution was placed in a 4 mL vial with a penetrable cap, sealed with electrical tape, and also removed from the glovebox. The NMR sample was locked, tuned, shimmed, and its signal was measured following the standard procedure. Afterward, the sample was removed from the instrument. Subsequently, 30 µL of the G3 solution was added to the NMR tube, which was shaken vigorously. The tube was placed back into the instrument, and its signal was measured every 30 seconds using the multizg function. The concentration of the CC was measured relative to the internal standard, 1,4-bis(trimethylsilyl)benzene.

3.3. Van't Hoff Analysis

A G3 solution was prepared by dissolving 14.5 mg of G3 and 7.3 mg of benzoquinone in 500 µL of $CDCl_3$ (without 1,4-bis(trimethylsilyl)benzene). 600 µL of the CC stock solution was transferred to a J-Young NMR tube, followed by the addition of 30 µL of the G3 solution. The tube was then sealed tightly, shaken vigorously, and removed from the glovebox. Equilibrium concentrations were measured sequentially at -20 °C, 10 °C, 25 °C, 40 °C, and 55 °C using the same sample. The concentration of the CC was measured relative to the internal standard, 1,4-bis(trimethylsilyl)benzene. At each temperature, the sample was tuned and shimmed before measurement. Signals were collected every 30 seconds until the concentration plateaued for at least 10 minutes. The average concentration during this plateau was recorded as the equilibrium concentration for each temperature.

3.4. Kinetic Experiments

3.4.1. Generation of first dataset

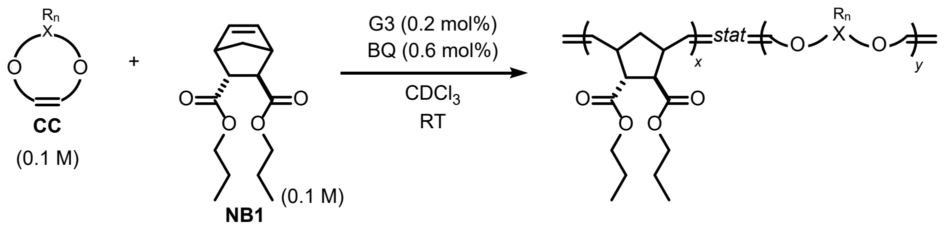

300 µL of the CC stock solution and 300 µL of the **NB1** stock solution were transferred to an NMR tube, sealed with a penetrable rubber septum and electrical tape, then removed from the glovebox. A G3 solution was prepared fresh by dissolving 14.5 mg of G3 and 7.3 mg of benzoquinone in 1,000 µL of $CDCl_3$. The G3 solution was placed in a 4 mL vial with a penetrable cap, sealed with electrical tape, and also removed from the glovebox. The NMR sample was locked, tuned, shimmed, and its initial state signal was measured following standard procedures. Afterward, the sample was removed from the instrument. Subsequently, 12 µL of the G3 solution was added to the NMR tube, which was shaken vigorously for a few seconds. The tube was immediately placed back into the instrument, and its signal was measured every 30 seconds using the multizg function. The concentration of the monomers was measured relative to the internal standard, 1,4-bis(trimethylsilyl)benzene.

3.4.2. Generation of second dataset

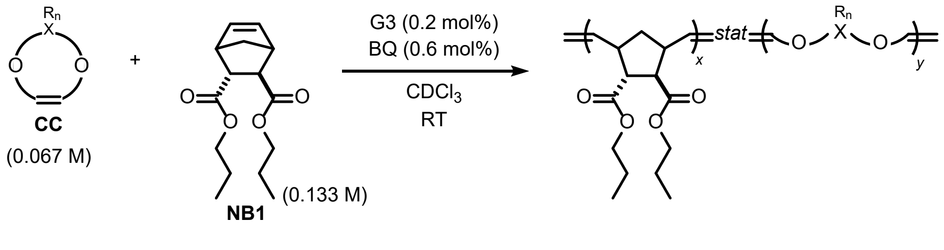

200 µL of the CC stock solution and 400 µL of the **NB1** stock solution were transferred to an NMR tube. The remaining procedure followed the steps outlined in section 3.4.1.

3.4.3. Generation of third dataset

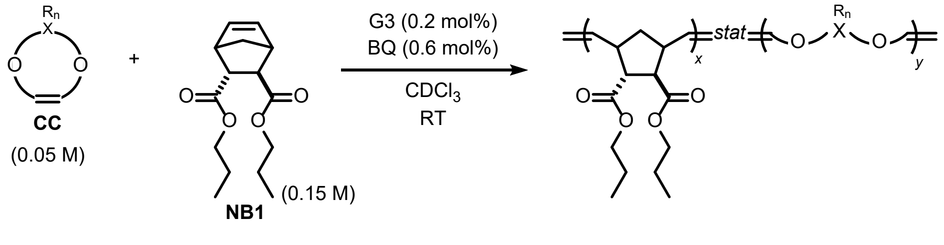

150 µL of the CC stock solution and 450 µL of the **NB1** stock solution were transferred to an NMR tube. The remaining procedure followed the steps outlined in section 3.4.1.

3.5. Copolymer Synthesis

3.5.1. Synthesis of copolymers **p(NB1-*co*-CC)**s

A G3 stock solution was prepared fresh by dissolving 14.5 mg of G3 and 7.3 mg of benzoquinone in 1,000 µL of $CDCl_3$. 570 µL of the **NB1** stock solution and 30 µL of the CC stock solution were transferred to an NMR tube. After thoroughly mixing the solutions, 12 µL of the G3 stock was added using a microsyringe, followed by vigorous mixing. The polymerization was conducted at room temperature for 90 minutes. Subsequently, 100 µL of ethyl vinyl ether (EVE) was added to quench the polymerization. The resulting crude mixture was transferred to a 20 mL vial, and the volatiles were removed in vacuo.

3.5.2. Synthesis of copolymer **p(NB1-*co*-Me_4Si_2O9)'**

A G3 stock solution was prepared fresh by dissolving 14.5 mg of G3 in 1,000 µL of $CDCl_3$. 570 µL of the **NB1** stock solution and 30 µL of **Me_4Si_2O9** stock solution were transferred to an NMR tube. After thoroughly mixing the solutions, 12 µL of the G3 stock was added using a microsyringe, followed by vigorous mixing. The polymerization was conducted at room temperature for 90 minutes. Subsequently, 100 µL of EVE was added to quench the polymerization. The resulting crude mixture was transferred to a 20 mL vial, and the volatiles were removed in vacuo.

3.5.3. Synthesis of homopolymers **pNB1**, **pNB2**, and **pNB3**

A G3 stock solution was prepared fresh by dissolving 14.5 mg of G3 in 1,000 µL of $CDCl_3$. 600 µL of the NBE stock solution was transferred to an NMR tube. After thoroughly mixing the solutions, 12 µL of the G3 stock was added using a microsyringe, followed by vigorous mixing. The polymerization was conducted at room temperature for 90 minutes (for **NB1**), 40 minutes (for **NB2**), or 36 hours (for **NB3**). Subsequently, 100 µL of EVE was added to quench the polymerization. The resulting crude mixture was transferred to a 20 mL vial, and the volatiles were removed in vacuo.

3.5.4. Synthesis of copolymers **p(NB2-*co*-Me_4Si_2O9)** and **p(NB3-*co*-Me_4Si_2O9)**

A G3 stock solution was prepared fresh by dissolving 14.5 mg of G3 in 1,000 µL of $CDCl_3$. 570 µL of the NBE stock solution and 30 µL of **Me_4Si_2O9** stock solution were transferred to an NMR tube. After thoroughly mixing the solutions, 12 µL of the G3 stock was added using a microsyringe, followed by vigorous mixing. The polymerization was conducted at room temperature for 40 minutes (for **NB2**) or 36 hours (for **NB3**). Subsequently, 100 µL of EVE was added to quench the polymerization. The resulting crude mixture was transferred to a 20 mL vial, and the volatiles were removed in vacuo.

3.5.5. Synthesis of polymers **p(NB4-*co*-Me_4Si_2O9)** and **pNB4**

A 40 mM stock solution of **NB4** was prepared by dissolving 135.1 mg of **NB4** in 1,000 μL of $CDCl_3$. A 40 mM stock solution of **Me_4Si_2O9** was prepared by diluting the 200 mM stock solution 1:5 with $CDCl_3$. A G3 stock solution was freshly prepared by dissolving 7.3 mg of G3 in 1,000 μL of $CDCl_3$. **p(NB4-co-Me_4Si_2O9)** was synthesized by mixing 200 μL of the 40 mM **NB4** stock with 50 μL of the 40 mM **Me_4Si_2O9** stock, followed by the addition of 10 μL of the G3 stock and immediate vortexing. **pNB4** was synthesized by diluting 200 μL of the 40 mM **NB4** stock with 50 μL of $CDCl_3$, then adding 10 μL of the G3 stock and immediately vortexing. The polymerizations were run for 1 hour, after which 100 μL of EVE was added to quench the reaction. Each resulting mixture was transferred to a separate 20 mL vial, and the volatiles were removed in vacuo.

3.6. Copolymer Deconstruction

3.6.1. Deconstruction rate comparison

3.6.1.1 Acidic deconstruction

A solution of 10^{-4} M HCl in 1:1 (v/v) mixture of 1-propanol and 1,4-dioxane was added to the copolymer, previously dried in vacuo, to achieve a final concentration of 5 mg/mL. The resulting solution was stirred at room temperature, and aliquots were collected at predetermined time points. Each aliquot was immediately quenched by addition to an excess of solid sodium bicarbonate, sonicated for 20 seconds, and allowed to stand for 5 minutes. The supernatant was then transferred and concentrated in vacuo, and the resulting product was analyzed by SEC.

3.6.1.2. Complete acidic deconstruction (for reference)

A solution of 2 M HCl in 1:1 (v/v) 1-propanol:1,4-dioxane was added to the copolymer, previously dried in vacuo, to achieve a final concentration of 5 mg/mL. The resulting mixture was stirred at room temperature for 1 hour and then quenched by addition to an excess of solid sodium bicarbonate. The mixture was sonicated for 20 seconds and allowed to stand for 5 minutes. The supernatant was subsequently transferred and concentrated in vacuo, and the resulting product was analyzed by SEC.

3.6.1.3. Quantification of polymer deconstruction

The extent of polymer deconstruction was calculated based on a previously reported method (*Nat. Chem.* **2019**, *11*, 1124–1132.), using the fully deconstructed counterpart as a reference.

3.6.2. Alcoholysis

1 mL of anhydrous ^{n}PrOH was added to the vial containing the polymer, followed by the addition of 1 mL of 4M HCl in 1,4-dioxane. The resulting mixture was stirred at 50 °C for 6 hours. Afterward, the solvent was removed under high vacuum, and the residue was analyzed by SEC.

3.6.3. Hydrolysis

2 mL of THF was added to the vial containing the polymer, followed by the addition of 0.5 mL of 1 M HCl solution in water. The resulting mixture was stirred at room temperature for 6 hours. Anhydrous sodium sulfate was then added to the vial, and the mixture was vortexed for 1 minute before being filtered through a 0.2 μm syringe filter. 2 mL of DCM was used to minimize the loss of polymer fragments. The solvent was removed under reduced pressure, and the residue was analyzed by SEC.

3.7. Safety Statement

No unexpected or unusually high safety hazards were encountered while carrying out the procedures described in Section 3.

4. Synthesis and Characterization of Monomers

4.1. Synthesis of Me_4Si_28

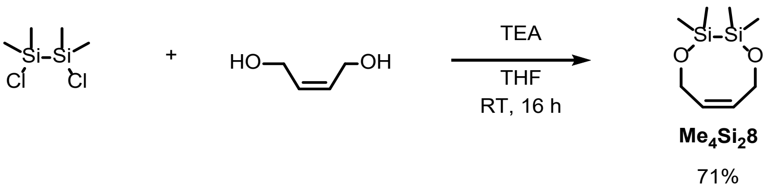

To a flame-dried, nitrogen-charged 1000 mL round-bottom flask equipped with a magnetic stir bar, 600 mL of anhydrous THF and 33.5 mL of anhydrous TEA (240 mmol, 2.0 equiv) were added. In a separate flame-dried, nitrogen-charged 50 mL flask, 9.86 mL of cis-2-butene-1,4-diol (120 mmol, 1.0 equiv) was added and then diluted with anhydrous THF to a total volume of 22.4 mL. This solution was transferred into a 24 mL syringe. Another 24 mL syringe was charged with 22.4 mL of 1,2-dichlorotetramethyldisilane (120 mmol, 1.0 equiv). The contents of the two syringes were added simultaneously, dropwise, into the 1000 mL flask over 10 hours at room temperature. After the addition was complete, the reaction mixture was stirred for an additional 6 hours at room temperature.

The reaction mixture was then filtered through a fritted glass funnel to remove the triethylammonium salt. The filter cake was washed with approximately 100 mL of hexanes to minimize product loss. The filtrate was concentrated using a rotary evaporator, transferred to a 1000 mL separatory funnel, and diluted with 250 mL of hexanes. The resulting organic layer was washed with 250 mL of water (once) and 250 mL of brine (twice), then dried over anhydrous Na_2SO_4, and concentrated in vacuo. Vacuum distillation of the crude mixture yielded the desired product as a colorless oil (17.2 g, 71% yield).

^{1}H NMR (400 MHz, $CDCl_3$) δ 5.97 (td, J = 5.0, 5.0, 2.5 Hz, 2H), 4.39 – 4.19 (m, 4H), 0.22 (s, 12H).

^{13}C{^{1}H} NMR (101 MHz, $CDCl_3$) δ 132.20, 58.95, 0.37.

HRMS (DART-TOF) $C_8H_{19}O_2Si_2$ $[M+H]^+$ calcd: 203.09181 found: 203.09208

4.2. Synthesis of Me_4Si_2O9

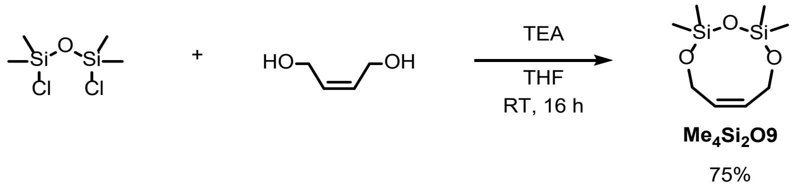

To a flame-dried, nitrogen-charged 1000 mL round-bottom flask equipped with a magnetic stir bar, 800 mL of anhydrous THF and 30.7 mL of anhydrous TEA (220 mmol, 2.0 equiv) were added. In a separate flame-dried, nitrogen-charged 50 mL flask, 9.04 mL of cis-2-Butene-1,4-diol (110 mmol, 1.0 equiv) was added and then diluted with anhydrous THF to a total volume of 21.5 mL. This solution was transferred into a 24 mL syringe. Another 24 mL syringe was charged with 21.5 mL of 1,3-dichloro-1,1,3,3-tetramethyldisiloxane (110 mmol, 1.0 equiv). The contents of the two syringes were added simultaneously, dropwise, into the 1000 mL flask over 10 hours at room temperature. After the addition was complete, the reaction mixture was stirred for an additional 6 hours at room temperature.

The reaction mixture was then filtered through a fritted glass funnel to remove the triethylammonium salt. The filter cake was washed with approximately 100 mL of hexanes to minimize product loss. The filtrate was concentrated using a rotary evaporator, transferred to a 1000 mL separatory funnel, and diluted with 250 mL of hexanes. The resulting organic layer was washed with 250 mL of water (once) and 250 mL of brine (twice), then dried over anhydrous Na_2SO_4, and concentrated in vacuo. Vacuum distillation of the crude mixture yielded the desired product as a colorless oil (18.1 g, 75% yield).

^{1}H NMR (400 MHz, $CDCl_3$) δ 5.93 – 5.76 (m, 2H), 4.46 – 4.27 (m, 4H), 0.12 (s, 12H).

^{13}C{^{1}H} NMR (101 MHz, $CDCl_3$) δ 130.86, 57.82, 0.24.

HRMS (DART-TOF) $C_8H_{19}O_3Si_2$ $[M+H]^+$ calcd: 219.08672 found: 219.08659

4.3. Synthesis of iPr_4Si_2O9

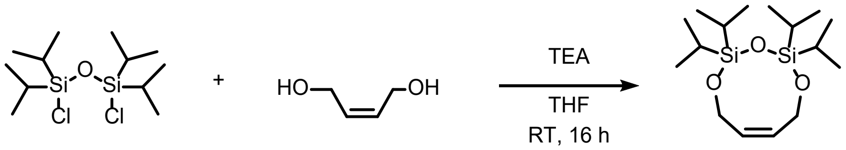

iPr_4Si_2O9

69%

To a flame-dried, nitrogen-charged 500 mL round-bottom flask equipped with a magnetic stir bar, 200 mL of anhydrous THF and 7 mL of anhydrous TEA (50 mmol, 2.0 equiv) were added. In a separate flame-dried, nitrogen-charged 50 mL flask, 2.1 mL of cis-2-butene-1,4-diol (25 mmol, 1.0 equiv) was added and diluted with anhydrous THF to a total volume of 12 mL. This solution was transferred to a 12 mL syringe. Another 12 mL syringe was charged with 8.0 mL of 1,3-dichloro-1,1,3,3-tetraisopropyldisiloxane (25 mmol, 1.0 equiv) and diluted with THF to a total volume of 12 mL. The contents of both syringes were added simultaneously, dropwise, into the 500 mL flask over 4 hours at room temperature. After the addition was complete, the reaction mixture was stirred for an additional 12 hours at room temperature.

The reaction mixture was then filtered through a fritted glass funnel to remove the triethylammonium salts. The filter cake was washed with approximately 50 mL of hexanes to minimize product loss. The filtrate was concentrated using a rotary evaporator, transferred to a 500 mL separatory funnel, and diluted with 100 mL of hexanes. The resulting organic layer was washed with 100 mL of water (once) and 100 mL of brine (twice), then dried over anhydrous Na_2SO_4 and concentrated in vacuo. Vacuum distillation of the crude mixture yielded the desired product as a viscous, colorless oil (5.7 g, 69% yield).

^{1}H NMR (400 MHz, $CDCl_3$) δ 5.90 – 5.73 (m, 2H), 4.48 – 4.28 (m, 4H), 1.17 – 0.87 (m, 28H).

^{13}C{^{1}H} NMR (101 MHz, $CDCl_3$) δ 130.74, 57.86, 17.53, 17.40, 13.59.

HRMS (DART-TOF) $C_{16}H_{35}O_3Si_2$ $[M+H]^+$ calcd: 331.21192 found: 331.21440

4.3. Synthesis of Me2Si7

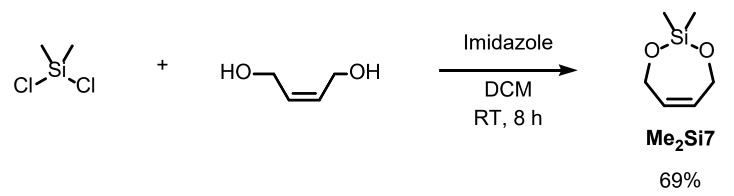

The compound was synthesized following a modified procedure from the literature.[7]

To a flame-dried 1000 mL round-bottom flask equipped with a magnetic stir bar, 27.2 g of imidazole (400 mmol, 2.0 equiv) was added, after which the flask was sealed and nitrogen-charged. 800 mL of anhydrous DCM was then added. In a separate flame-dried, nitrogen-charged 50 mL flask, 16.4 mL of cis-2-butene-1,4-diol (200 mmol, 1.0 equiv) was added and diluted with anhydrous DCM to a total volume of 24.1 mL. This solution was transferred to a syringe. Another syringe was charged with 24.1 mL of dichlorodimethylsilane (200 mmol, 1.0 equiv). The contents of both syringes were added simultaneously, dropwise, into the 1000 mL flask over 4 hours at room temperature. After the addition was complete, the reaction mixture was stirred for an additional 4 hours at room temperature.

The reaction mixture was then filtered through a fritted glass funnel to remove the imidazolium salts. The filter cake was washed with approximately 50 mL of DCM to minimize product loss. The filtrate was concentrated using a rotary evaporator. Vacuum distillation of the crude mixture yielded the desired product as a colorless oil (19.9 g, 69% yield).

^{1}H NMR and $^{13}C\{^{1}H\}$ NMR spectra corresponded to those reported in the literature.[7]

4.4. Safety Statement

No unexpected or unusually high safety hazards were encountered while carrying out the procedures described in Section 4.

5. Fitting Details

5.1. Izu-Lundberg Equation

The experimental data were fitted to the following Izu-Lundberg equilibrium copolymer equation, originally reported by Izu and coworkers[8] and rectified by Lundberg and coworkers[9]:

$$\frac{d[A]}{d[B]} = -\frac{ak_1[A] + bk_5[A] - a[(1-\epsilon)k_6 + \epsilon k_2]}{bk_7[B] + ak_3[B] - b[(1-\eta)k_4 + \eta k_8]}$$

$$a + b = 1$$

$$a[k_3[B] + (1-\epsilon)k_6] = b[k_5[A] + (1-\eta)k_4]$$

$$b\epsilon(1-\eta)k_4 = a[\epsilon(k_1[A] + k_2 + k_3[B]) - (k_1[A] + \epsilon^2 k_2)]$$

$$a\eta(1-\epsilon)k_6 = b[\eta(k_7[B] + k_8 + k_5[A]) - (k_7[B] + \eta^2 k_8)]$$

k_2 and k_6 were set to 0, assuming irreversible propagation of NBEs.

5.2. Method Development

To accurately determine the kinetic and thermodynamic parameters that best represent the observed reaction behavior, we developed a method that combines an ordinary differential equation (ODE) with a set of coupled algebraic equations—the Izu-Lundberg equations. Two distinct experimental datasets were used to ensure reliable parameter estimation. The model simulates the conversion profiles of two species (M and CC) in the copolymerization process by calculating total conversion variables from the initial experimental conditions. The evolution of a fractional conversion variable is governed by an ODE, which we integrated numerically using SciPy's solve_ivp function. Simultaneously, the Izu-Lundberg equations were solved with the least-squares optimization routine available in scipy.optimize.least_squares. Residuals were calculated as the differences between the model-predicted conversion values and the corresponding experimental data for both species. These residuals formed the objective function in our nonlinear least-squares optimization, which iteratively adjusted the kinetic parameters to minimize the sum of squared deviations. To ensure robustness and avoid convergence to local minima, the optimization was repeated several times with randomized initial guesses. All model fitting was executed in Python, and the predictions were visualized using Matplotlib to facilitate direct comparison between the simulated conversion trajectories and the experimental measurements.

5.3. Evaluation of the Method

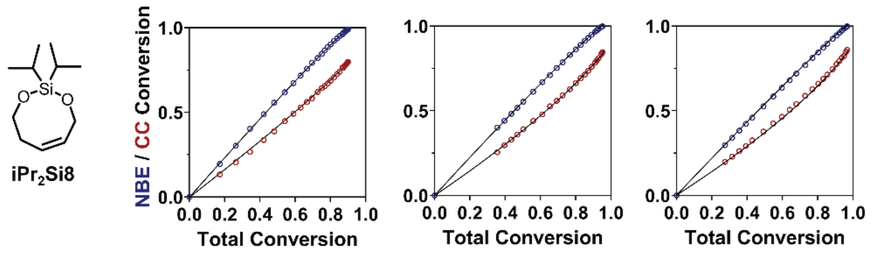

Figure S3. Evaluation of the method using **iPr2Si8** as the model compound. Data fitting was performed on the two primary datasets (left and middle), and the parameters obtained were assessed based on their agreement with the validation dataset (right).

The model's ability to accurately extract kinetic and thermodynamic parameters from experimental copolymerization data was evaluated using **iPr2Si8** as the model comonomer. Two distinct datasets were generated according to the procedures described in Sections 3.4.1 and 3.4.2, while a separate validation set was prepared following the protocol in Section 3.4.3. The developed model was fitted to the two primary datasets. Using the resulting parameters, the ODE governing the evolution of a fractional conversion variable was numerically integrated over its defined range with solve_ivp (using the RK45 method), applying the initial conditions and parameters to compute predicted conversion trajectories for species M and CC. These fractional conversion values were then used to calculate the predicted conversion outputs, effectively simulating the reaction progress. Finally, the differences between the predicted values and the experimental conversion data from the validation set for both species were squared and summed to yield a single residual norm. This metric quantitatively measures how well the model—using parameters fitted to the primary datasets—predicts the behavior observed in the validation set. The close agreement between the model predictions and the experimental data supports the robustness of the model in accurately determining the copolymerization parameters.

5.4. Fitting Results

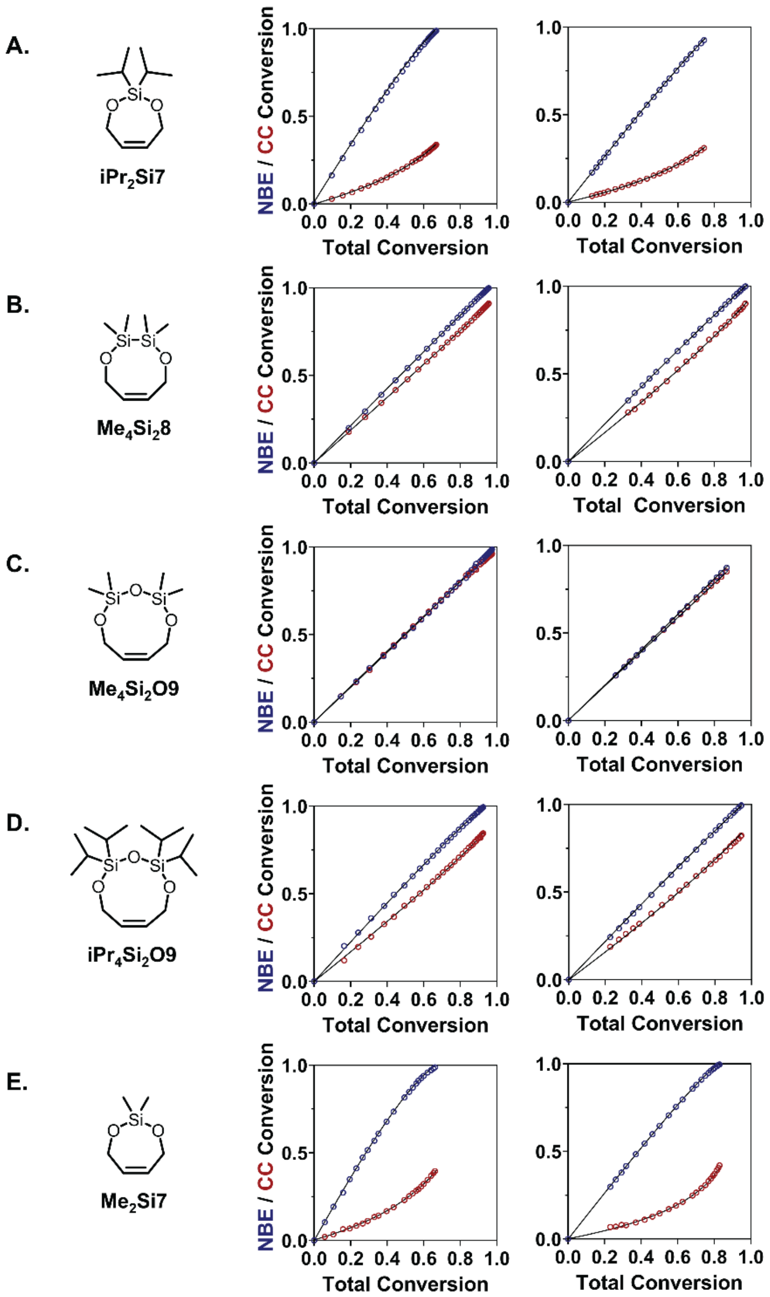

Figure S4. Application of the developed workflow to determine the copolymerization parameters. The figures display the fitting results for the two primary datasets (first dataset: left; second dataset: right).

6. Cost Estimation

The following cost analyses are based on prices listed by common chemical vendors as of December, 2024.

6.1. Me_4Si_2O9

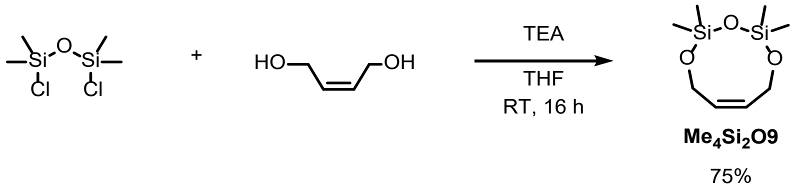

For the synthesis of 1 kg of **Me_4Si_2O9** (218.40 g/mol) with an overall yield of 75%, the cost estimation for the starting materials are as follows:

1. 1,3-Dichlorotetramethyldisiloxane
   - Required amount: 6.11 mol (203.21 g/mol)
   - Total weight: 1240.6 g
   - Price: $1532 for 2 kg (from Gelest)
   - Cost per synthesis: $950.3
2. cis-2-Butene-1,4-diol
   - Required amount: 6.11 mol (88.11 g/mol)
   - Total weight: 537.9 g
   - Price: $37 for 500 g (from Ambeed)
   - Cost per synthesis: $39.8
3. Triethylamine
   - Required amount: 12.2 mol (101.19 g/mol)
   - Total weight: 1235.53 g
   - Price: $785 for 40 kg (from Sigma-Aldrich)
   - Cost per synthesis: $24.3

Total Cost per 1 kg **Me_4Si_2O9** = $1014.4

6.2. iPr2Si8

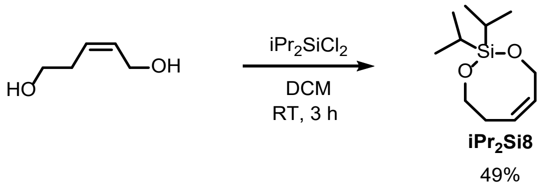

For the synthesis of 1 kg of **iPr2Si8** (214.38 g/mol) with an overall yield of 49%,[4] the cost estimation for the starting materials is as follows:

1. Imidazole
   - Required amount: 19.0 mol (68.077 g/mol)
   - Total weight: 1296.2 g
   - Price: $94 for 500 g (from Ambeed)
   - Cost per synthesis: $243.7
2. Diisopropyldichlorosilane
   - Required amount: 9.52 mol (185.17 g/mol)
   - Total weight: 1762.75 g
   - Price: $1670 for 1 kg (from Gelest)
   - Cost per synthesis: $2943.8
3. cis-2-Pentene-1,5-diol
   - Required amount: 9.52 mol
   - Cost per synthesis: $26,162.9 (see below for cost analysis)

Total Cost per 1 kg **iPr2Si8** = $29,350.4

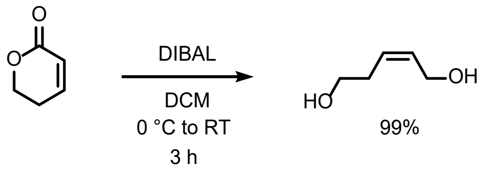

For the synthesis of 9.52 mol of cis-2-pentene-1,5-diol with an overall yield of 99%,[5] the cost estimation for the starting materials is as follows:

1. 5,6-Dihydro-2H-pyran-2-one
   - Required amount: 9.62 mol (98.10 g/mol)
   - Total weight: 943.33 g
   - Price: $600 for 25 g (from Ambeed)
   - Cost per synthesis: $22,639.9
2. Diisobutylaluminum hydride
   - Required amount: 24.05 mol (142.22 g/mol)
   - Total weight: 3420.4 g
   - Price: $1030 for 1 kg (from Sigma-Aldrich)
   - Cost per synthesis: $3,523.0

Total Cost per 9.52 mol cis-2-Pentene-1,5-diol = $26,162.9

7. Supplementary Data and Discussions

7.1. Equilibrium Concentration Measurement

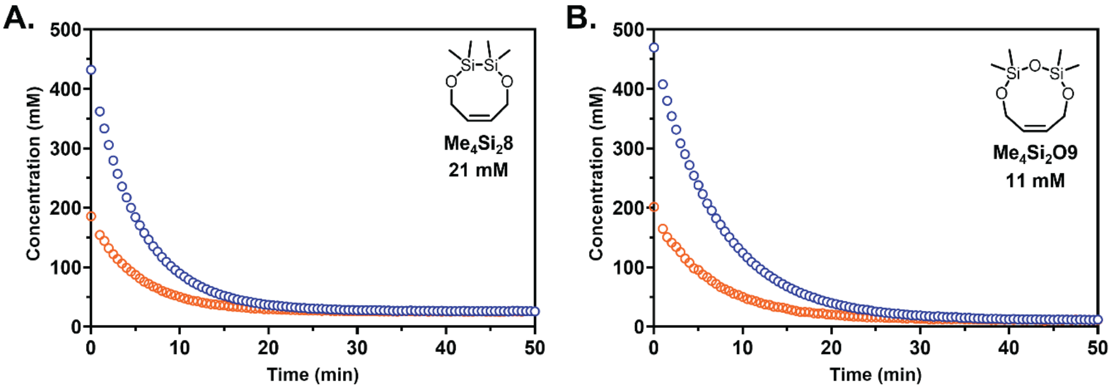

Figure S5. Equilibrium concentration measurement for (A) **Me4Si28** and (B) **Me4Si2O9**.

To support the assumption of negligible cyclic oligomer formation in determining equilibrium concentrations, equilibrium measurements were conducted for the newly developed eight-membered ring (**Me4Si28**) and nine-membered ring (**Me4Si2O9**) comonomers. Experiments were performed starting from two different initial concentrations (~200 mM and ~500 mM), both of which converged to final equilibrium values with no observable difference, consistent with minimal formation of cyclic oligomers.

7.2. Van't Hoff Analysis

In our Van't Hoff analysis, the standard enthalpy ($\Delta H°$) and entropy ($\Delta S°$) changes of polymerization were extracted from the slope (m) and intercept (b) of the linear regression of $ln\ K$ versus the reciprocal temperature ($1/T$).

An ordinary least-squares regression was performed using MATLAB's polyfit function, which returns the best-fit coefficients (m, b), the residual norm $||y - \hat{y}||^2 = \sqrt{\sum_i (y_i - \hat{y}_i)^2}$ (with $y_i = ln\ K_i$ and $\hat{y}_i = m/T_i + b$), and the upper-triangular R matrix. From these outputs, the residual variance was calculated as

$$\sigma^2 = \frac{||y - \hat{y}||^2}{n - 2} \tag{S1}$$

with n being the number of data points.

The covariance matrix of the fit coefficients then follows as

$$cov(m, b) = \sigma^2 R^{-1} (R^{-1})^T \tag{S2}$$

whose diagonal elements yield the variances σ_m^2 and σ_b^2. The standard errors $\sqrt{\sigma_m^2}$ and $\sqrt{\sigma_b^2}$ were converted into two-sided 95% confidence intervals

$$m \pm t_{0.975,n-2}\sqrt{\sigma_m^2}, \qquad b \pm t_{0.975,n-2}\sqrt{\sigma_b^2} \tag{S3}$$

where $t_{0.975,n-2}$ is the critical Student's t value for $n - 2$ degrees of freedom. Finally, the uncertainties in m and b were propagated linearly to $\Delta H°$ and $\Delta S°$.

Table S1. Data for Van't Hoff Analysis

		iPr_2Si7		iPr_2Si8		Me_4Si_28		iPr_4Si_2O9		Me_2Si7	
T (°C)	1/T (1/K)	$[M]_{eq}$ (M)	$ln(1/[M]_{eq})$	$[M]_{eq}$ (M)	$ln(1/[M]_{eq})$	$[M]_{eq}$ (M)	$ln(1/[M]_{eq})$	$[M]_{eq}$ (M)	$ln(1/[M]_{eq})$	$[M]_{eq}$ (M)	$ln(1/[M]_{eq})$
55	0.00305	0.1523	1.882	0.0550	2.900	0.0206	3.883	0.0468	3.062	0.0803	2.522
40	0.00319	0.1502	1.896	0.0498	2.999	0.0218	3.827	0.0444	3.115	0.0814	2.508
25	0.00335	0.1470	1.917	0.0481	3.034	0.0225	3.796	0.0420	3.170	0.0810	2.513
10	0.00353	0.1503	1.895	0.0433	3.140	0.0230	3.772	0.0413	3.186	0.0819	2.503
-20	0.00395	0.1508	1.892	0.0376	3.281	0.0242	3.722	0.0387	3.252	0.0830	2.489

The key compound, **Me_4Si_2O9**, was measured in six replicates (n = 6).

Table S2. Data for Van't Hoff Analysis of **Me_4Si_2O9**, Measured in Six Replicates

		**Me_4Si_2O9**											
T (°C)	1/T (1/K)	$[M]_{eq}$ (M)						$\ln(1/[M]_{eq})$					
55	0.00305	0.0129	0.0123	0.0123	0.0120	0.0120	0.0114	4.471	4.348	4.398	4.401	4.426	4.419
40	0.00319	0.0122	0.0117	0.0122	0.0128	0.0134	0.0112	4.490	4.410	4.449	4.410	4.361	4.310
25	0.00335	0.0125	0.0120	0.0123	0.0116	0.0111	0.0117	4.446	4.383	4.419	4.396	4.459	4.502
10	0.00353	0.0132	0.0117	0.0127	0.0108	0.0112	0.0110	4.510	4.331	4.452	4.368	4.526	4.489
-20	0.00395	0.0122	0.0111	0.0112	0.0104	0.0105	0.0104	4.565	4.406	4.497	4.492	4.566	4.560

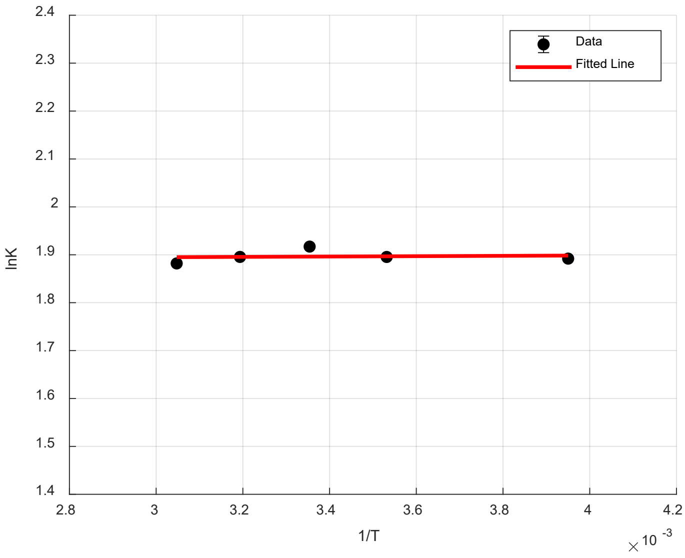

Figure S6. Van't Hoff analysis of **iPr_2Si7** (Slope: 3.60 ± 21.09, Intercept: 1.88 ± 0.07)

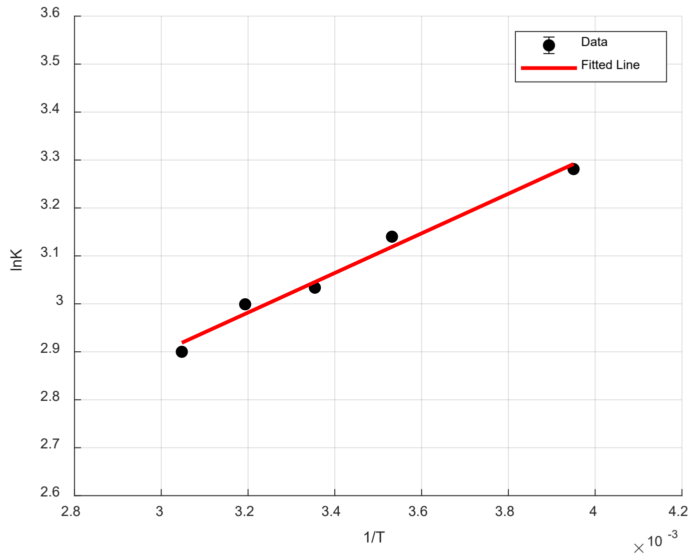

Figure S7. Van't Hoff analysis of **iPr_2Si8** (Slope: 412.96 ± 31.52, Intercept: 1.66 ± 0.11)

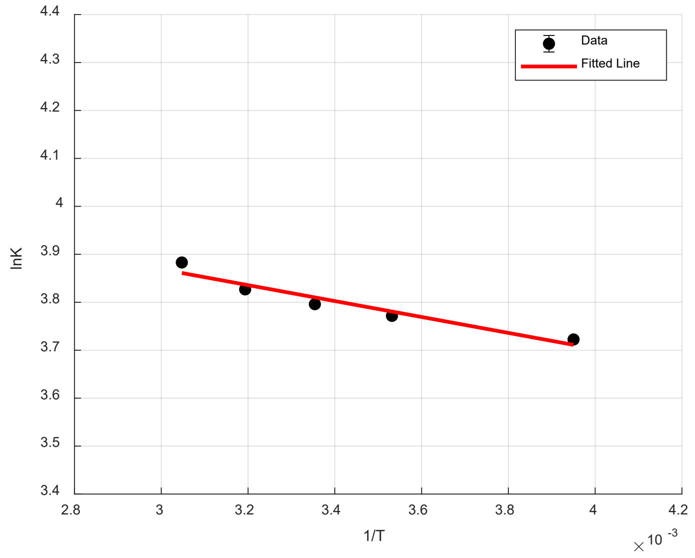

Figure S8. Van't Hoff analysis of **Me_4Si_28** (Slope: -166.05 ± 25.97, Intercept: 4.37 ± 0.09)

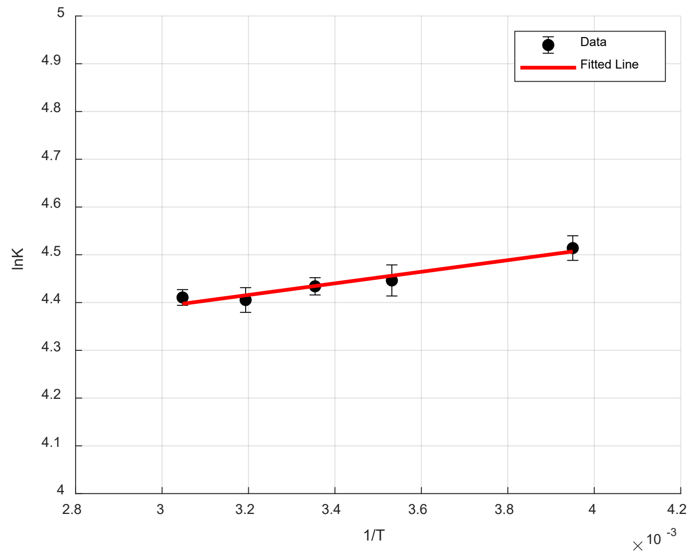

Figure S9. Van't Hoff analysis of **Me_4Si_2O9** (Slope: 121.28 ± 16.94, Intercept: 4.03 ± 0.06)

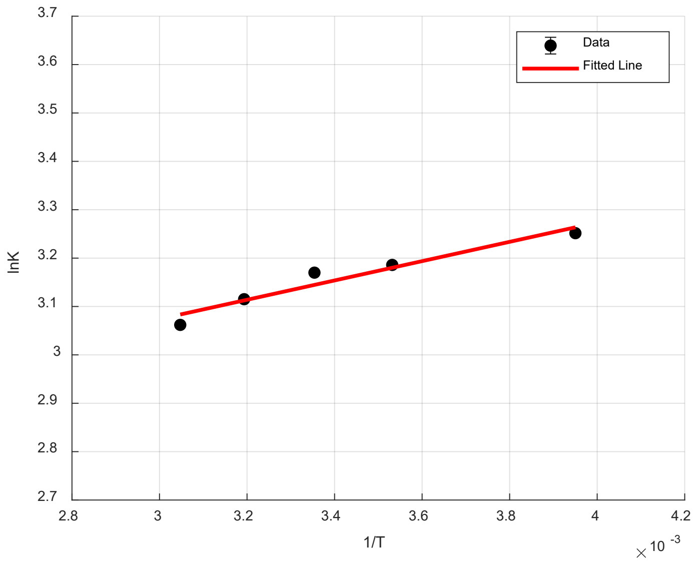

Figure S10. Van't Hoff analysis of **iPr_4Si_2O9** (Slope: 199.60 ± 29.66, Intercept: 2.48 ± 0.10)

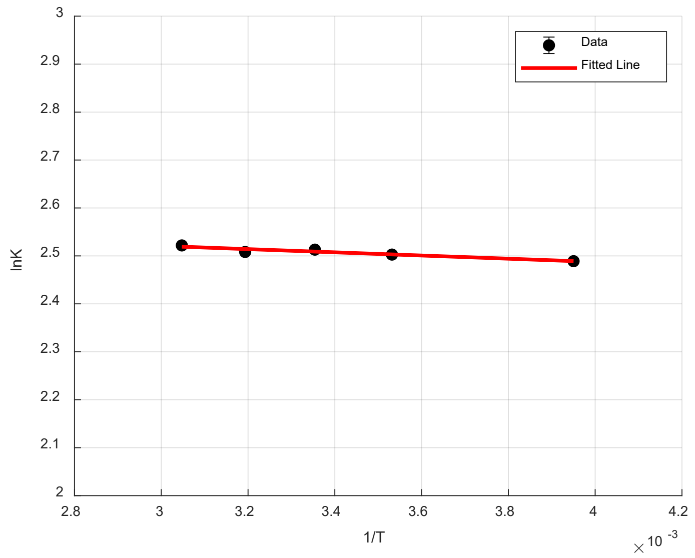

Figure S11. Van't Hoff Analysis of **Me$_2$Si7** (Slope: -33.25 ± 6.47, Intercept: 2.62 ± 0.02)

7.3. Copolymer Deconstruction Studies

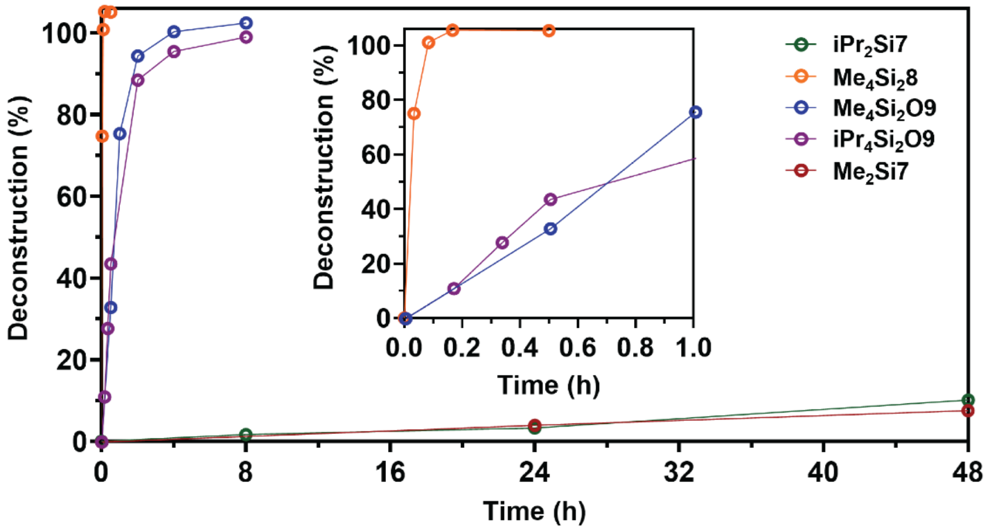

Figure S12. The percentage of backbone deconstruction as a function of time for **NB1**-CC copolymers incubated in 10^{-4} M HCl solution. The deconstruction rates followed the trend: **Me4Si28** < **Me2Si7** ≈ **Me4Si2O9** < **iPr2Si7** ≈ **iPr4Si2O9**. Inset: zoomed-in view showing differences in deconstruction rate between **Me4Si28**, **Me2Si7**, and **Me4Si2O9**.

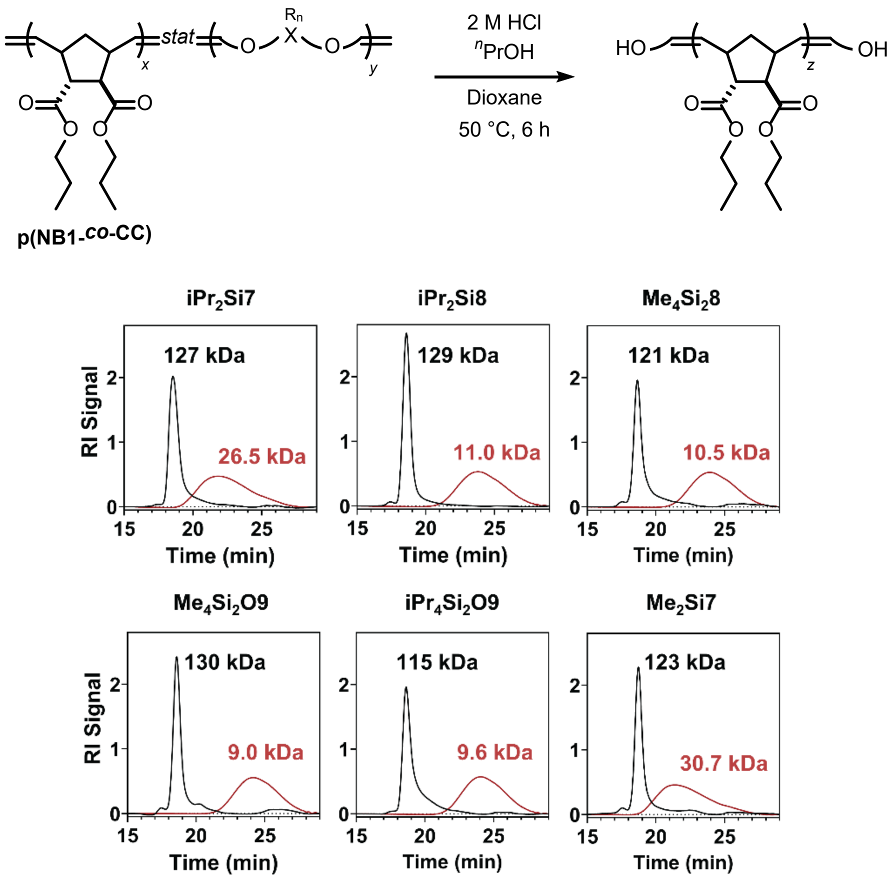

Figure S13. Evaluation of comonomer performance. The copolymers (black traces) and their deconstructed fragments (red traces) were analyzed via SEC using THF as the eluent. Molar masses from SEC were referenced against polystyrene standards, and M_w values are indicated in the figures.

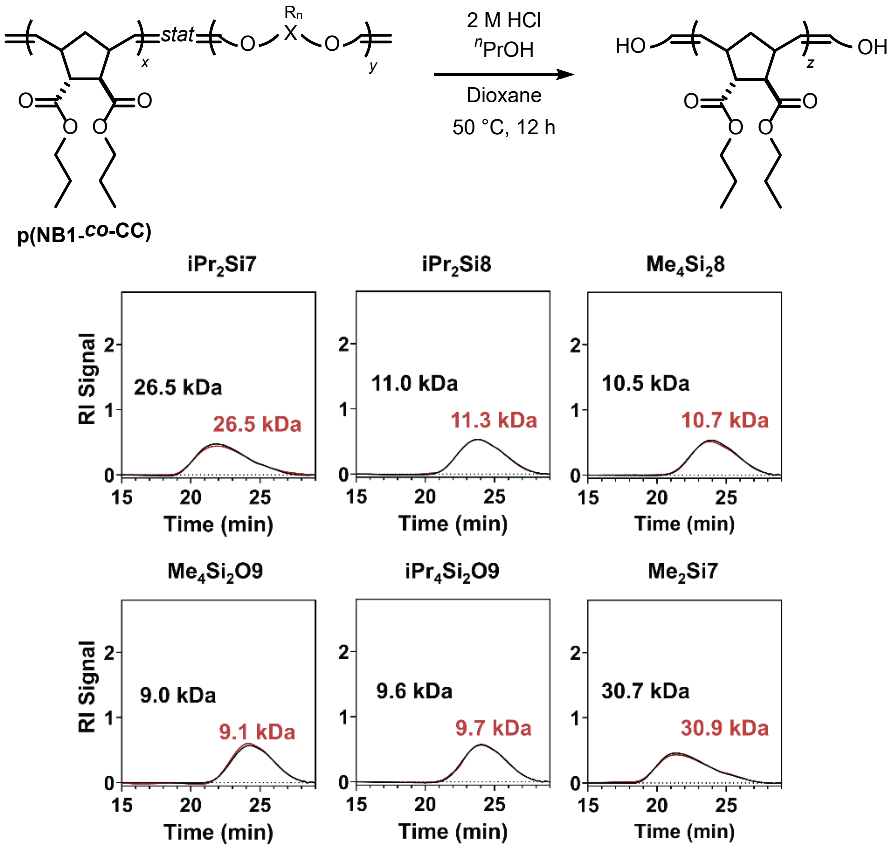

Figure S14. Confirmation of full deconstruction. The deconstruction duration was doubled to 12 hours, and the resulting fragments were analyzed via SEC using THF as the eluent (red traces). These traces were compared with those from a 6-hour deconstruction (black traces), showing no differences. Molar masses from SEC were referenced against polystyrene standards, and M_w values are indicated in the figures.

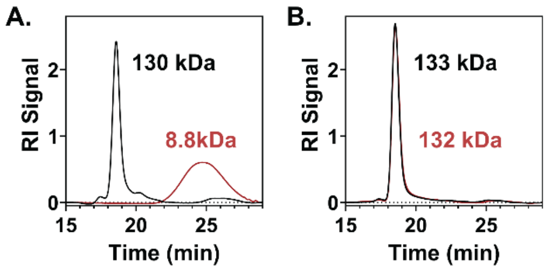

Figure S15. Hydrolysis of (A) **p(NB1-*co*-Me4Si2O9)** and (B) **pNB1**. THF was used as the eluent in SEC. The black traces correspond to the samples before deconstruction, while the red traces represent those after deconstruction. Molar masses were referenced against polystyrene standards, and M_w values are indicated in the figures.

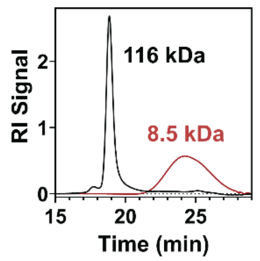

Figure S16. Alcoholysis of **p(NB1-*co*-Me4Si2O9)'**. THF was used as the eluent in SEC. The black traces correspond to the samples before deconstruction, while the red traces represent those after deconstruction. Molar masses were referenced against polystyrene standards, and M_w values are indicated in the figures

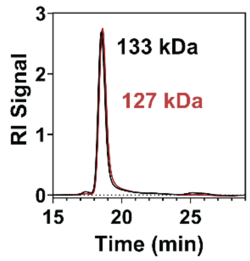

Figure S17. Alcoholysis of **pNB1**. THF was used as the eluent in SEC. The black traces correspond to the samples before deconstruction, while the red traces represent those after deconstruction. Molar masses were referenced against polystyrene standards, and M_w values are indicated in the figures

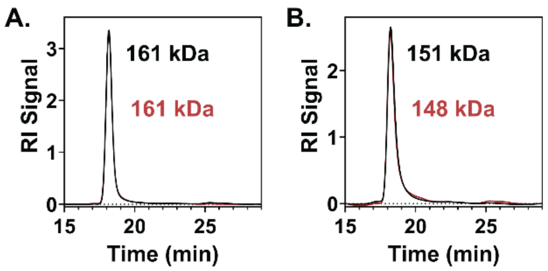

Figure S18. Hydrolysis of (A) **pNB2** (B) **pNB3**. THF was used as the eluent in SEC. The black traces correspond to the samples before deconstruction, while the red traces represent those after deconstruction. Molar masses were referenced against polystyrene standards, and M_w values are indicated in the figures

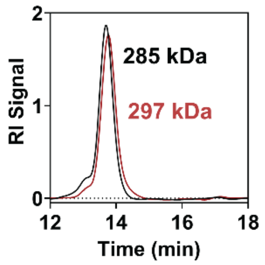

Figure S19. Hydrolysis of **pNB4**. DMF was used as the eluent in SEC. The black traces correspond to the samples before deconstruction, while the red traces represent those after deconstruction. Molar masses were determined using SEC-MALS, and M_w values are indicated in the figures.

8. NMR Spectra

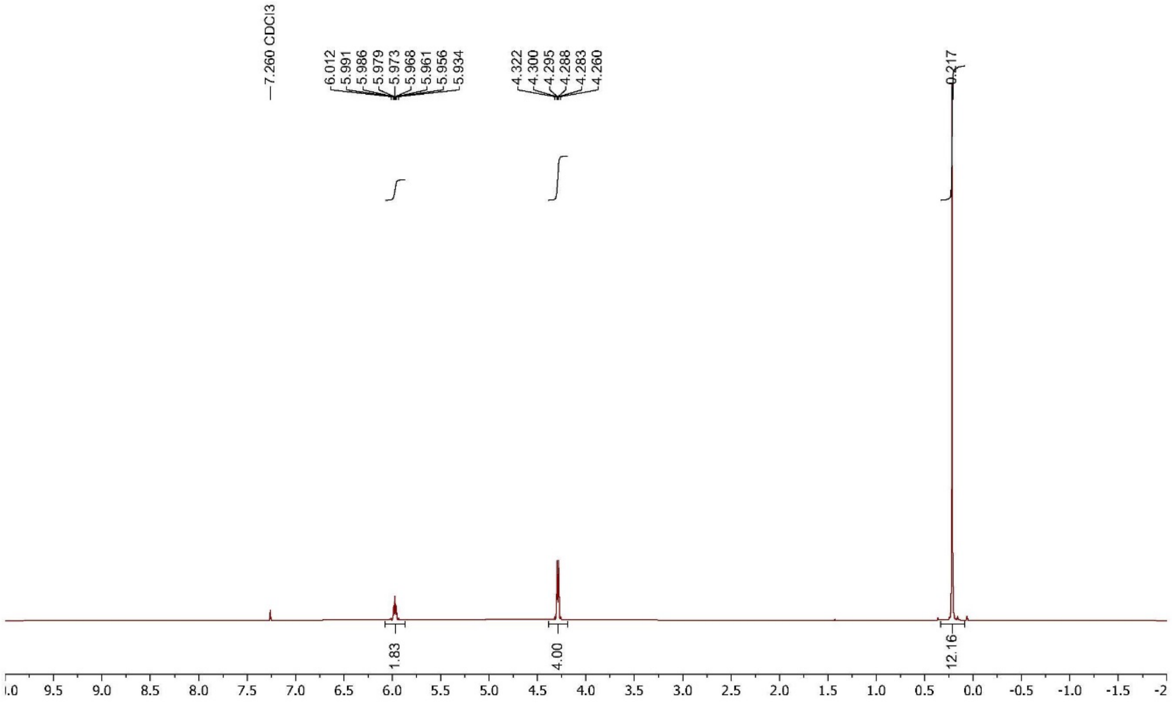

Figure S20. ^{1}H NMR (400 MHz, $CDCl_3$) spectrum of $\mathbf{Me_4Si_28}$

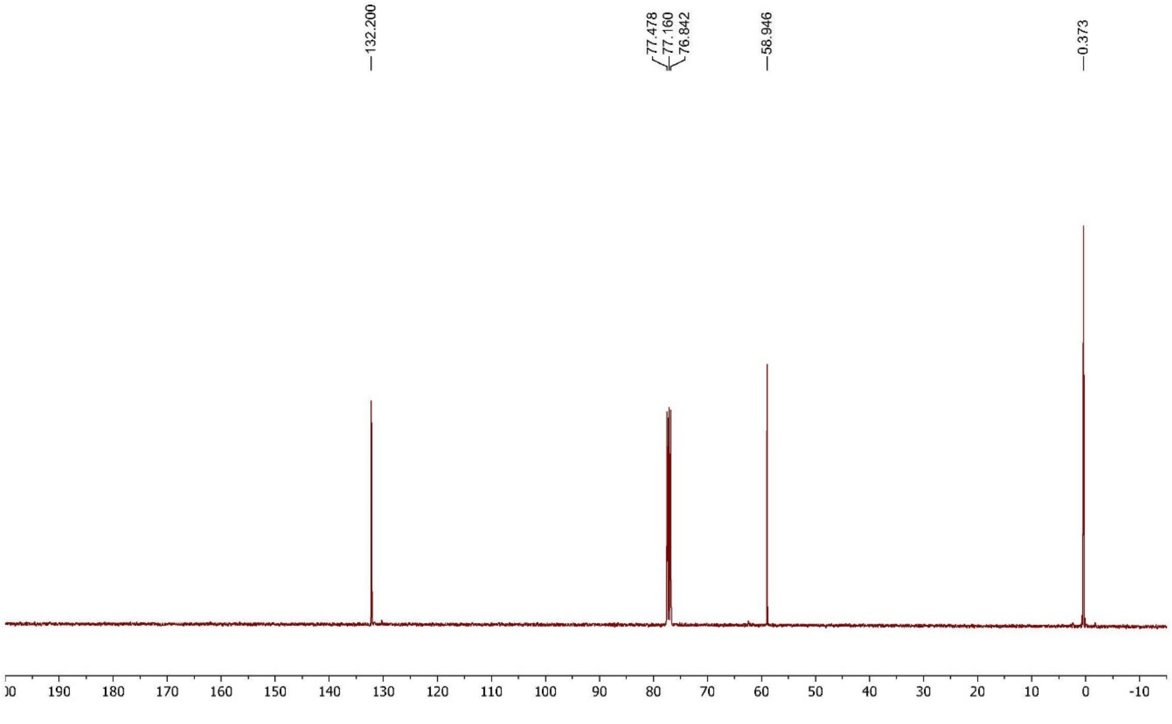

Figure S21. $^{13}C\{^{1}H\}$ NMR (101 MHz, $CDCl_3$) spectrum of $\mathbf{Me_4Si_28}$

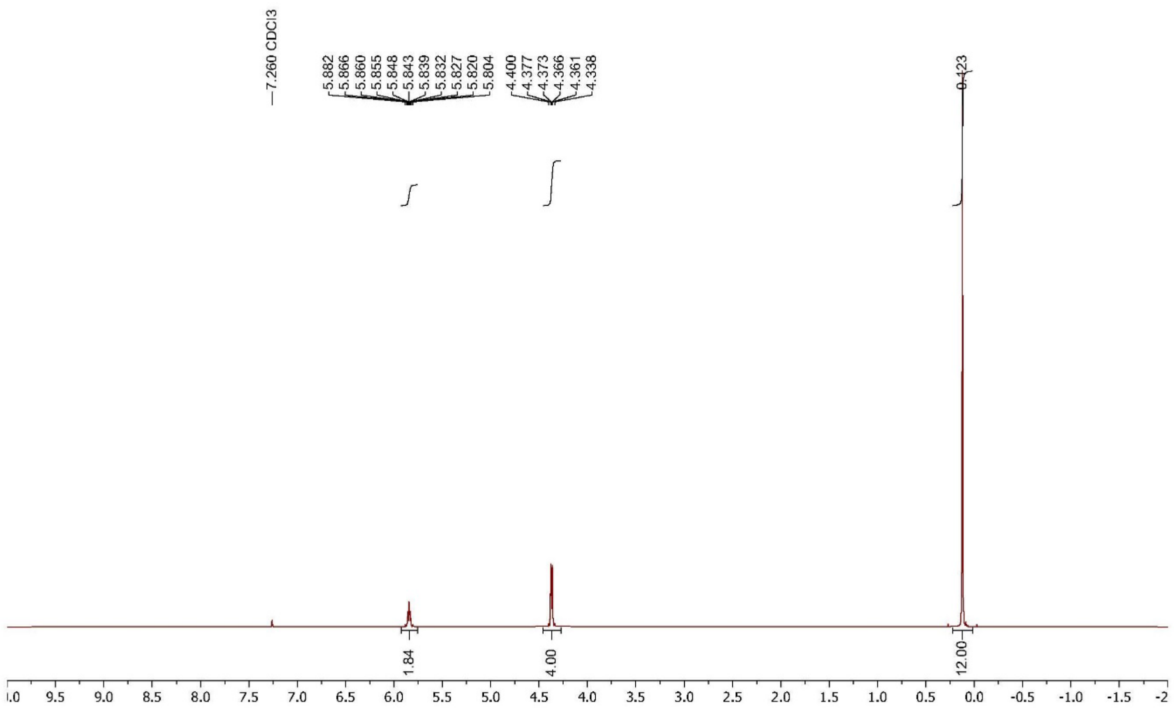

Figure S22. ^{1}H NMR (400 MHz, $CDCl_3$) spectrum of **Me_4Si_2O9**

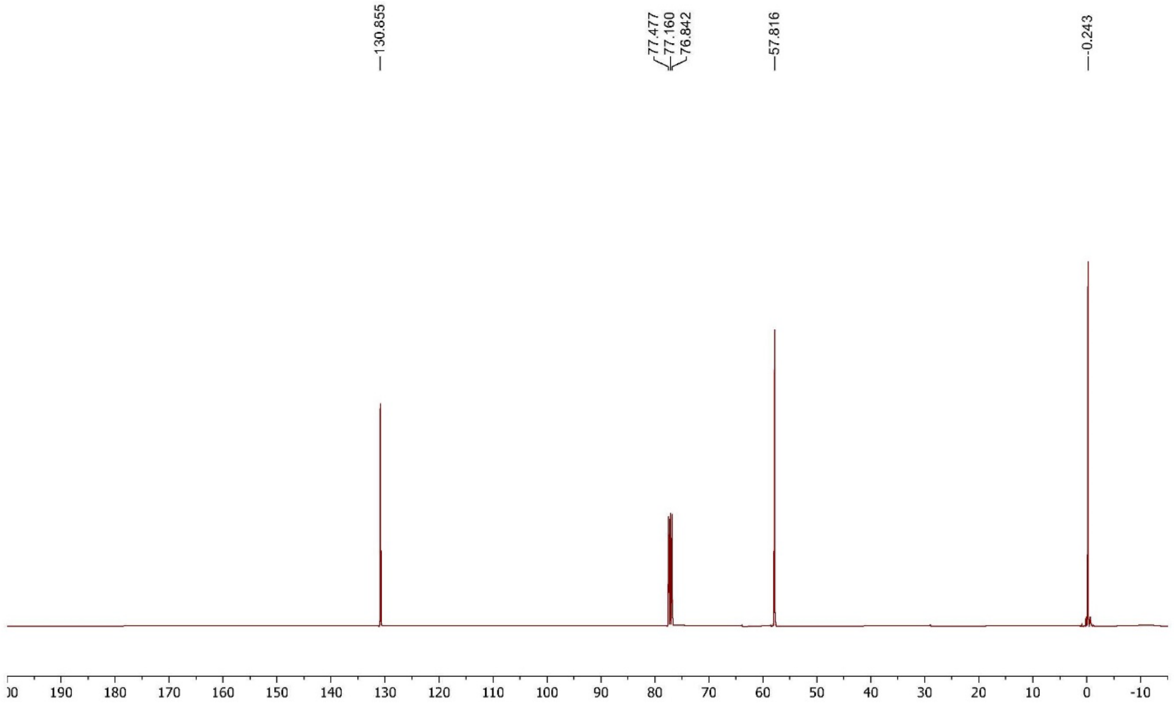

Figure S23. $^{13}C\{^{1}H\}$ NMR (101 MHz, $CDCl_3$) spectrum of **Me_4Si_2O9**

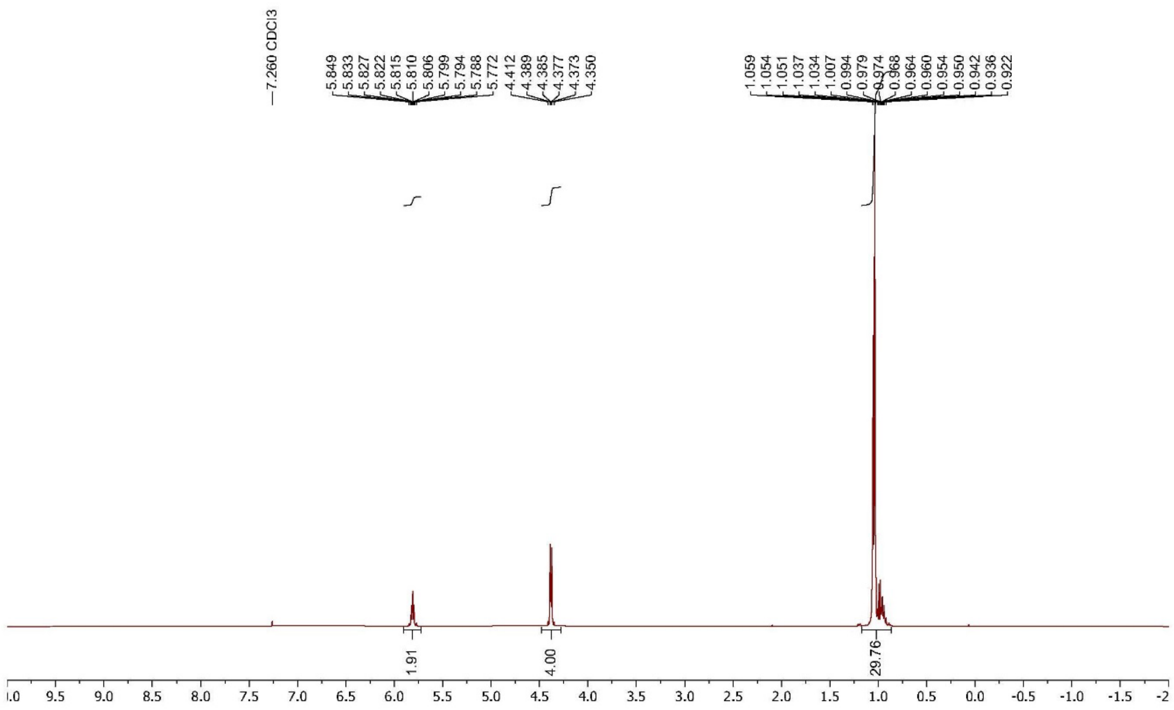

Figure S24. ^{1}H NMR (400 MHz, $CDCl_3$) spectrum of **iPr$_4$Si$_2$O9**

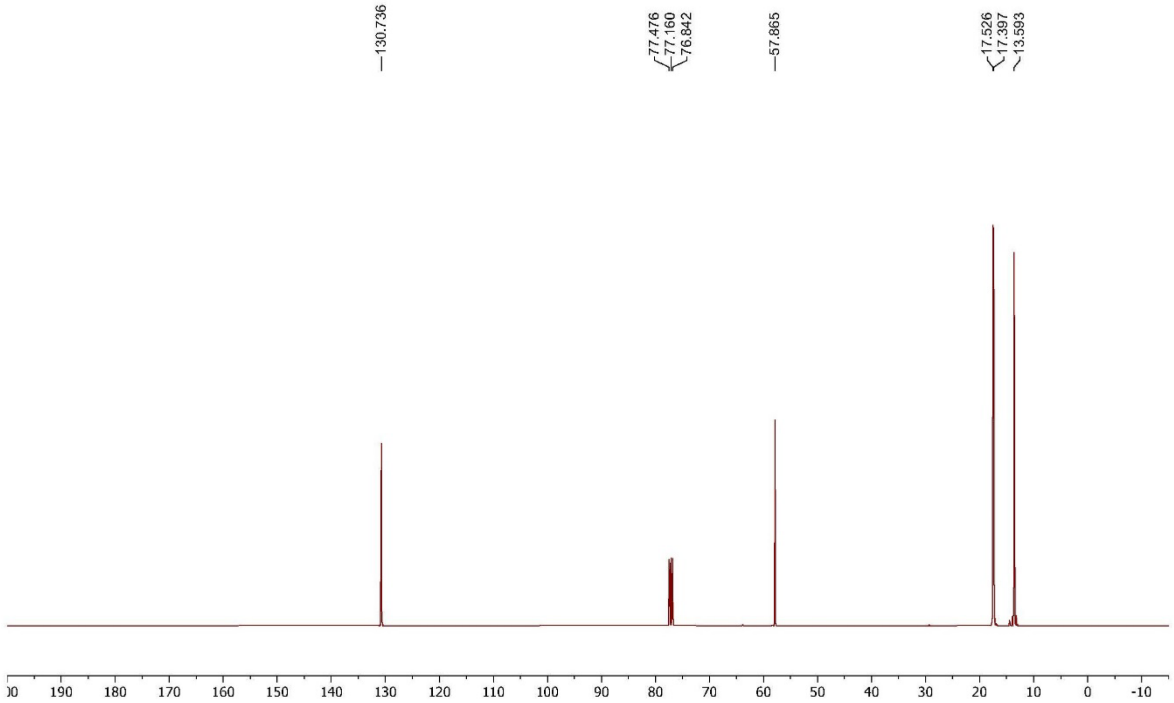

Figure S25. ^{13}C{^{1}H} NMR (101 MHz, $CDCl_3$) spectrum of **iPr$_4$Si$_2$O9**

8. References and Notes

(1) Sanford, M. S.; Love, J. A.; Grubbs, R. H. A Versatile Precursor for the Synthesis of New Ruthenium Olefin Metathesis Catalysts Melanie. *Organometallics* **2001**, *20*, 5314– 5318.

(2) Shieh, P.; Nguyen, H. V.-T.; Johnson, J. A. Tailored Silyl Ether Monomers Enable Backbone-Degradable Polynorbornene-Based Linear, Bottlebrush, and Star Copolymers through ROMP. *Nat. Chem.* **2019**, *11*(12), 1124–1132.

(3) Chang, A. B.; Lin, T.-P.; Thompson, N. B.; Luo, S.-X.; Liberman-Martin, A. L.; Chen, H.-Y.; Lee, B.; Grubbs, R. H. Design, Synthesis, and Self-Assembly of Polymers with Tailored Graft Distributions. *J. Am. Chem. Soc.* **2017**, *139*, 17683–17693.

(4) Chang, A. B.; Bates, F. S. Impact of Architectural Asymmetry on Frank–Kasper Phase Formation in Block Polymer Melts. *ACS Nano* **2020**, *14*, 11463–11472.

(5) Combe, C. M. S.; Biniek, L.; Schroeder, B. C.; McCulloch, I. Synthesis of [1]Benzothieno[3,2-b][1]Benzothiophene Pendant and Norbornene Random Co-Polymers via Ring Opening Metathesis. *J. Mater. Chem. C* **2014**, *2*, 538–541.

(6) Liu, J.; Burts, A. O.; Li, Y.; Zhukhovitskiy, A. V.; Ottaviani, M. F.; Turro, N. J.; Johnson, J. A. "Brush-First" Method for the Parallel Synthesis of Photocleavable, Nitroxide-Labeled Poly(Ethylene Glycol) Star Polymers. *J. Am. Chem. Soc.* **2012**, *134*(39), 16337–16344.

(7) Husted, K. E. L.; Brown, C. M.; Shieh, P.; Kevlishvili, I.; Kristufek, S. L.; Zafar, H.; Accardo, J. V.; Cooper, J. C.; Klausen, R. S.; Kulik, H. J.; Moore, J. S.; Sottos, N. R.; Kalow, J. A.; Johnson, J. A. Remolding and Deconstruction of Industrial Thermosets via Carboxylic Acid-Catalyzed Bifunctional Silyl Ether Exchange. *J. Am. Chem. Soc.* **2023**, *145*, 1916– 1923,

(8) Howell, J. A.; Izu, M.; O'Driscoll, K. F. Copolymerization with Depropagation. III. Composition and Sequence Distribution from Probability Considerations. *J. Polym. Sci. A1* **1970**, *8* (3), 699– 710.

(9) Lundberg, D. J.; Kilgallon, L. J.; Cooper, J. C.; Starvaggi, F.; Xia, Y.; Johnson, J. A. Accurate Determination of Reactivity Ratios for Copolymerization Reactions with Reversible Propagation Mechanisms. *Macromolecules* **2024**, *57* (14), 6727–6740.
